# Low-carbohydrate diets for type 1 diabetes mellitus: A systematic review

**DOI:** 10.1371/journal.pone.0194987

**Published:** 2018-03-29

**Authors:** Jessica L. Turton, Ron Raab, Kieron B. Rooney

**Affiliations:** 1 Discipline of Nutrition and Dietetics, School of Life and Environmental Sciences, Faculty of Science, University of Sydney, Sydney, NSW, Australia; 2 Insulin for Life Australia, Melbourne, Victoria, Australia; 3 Discipline of Exercise and Sport Science, Faculty of Health Sciences, Charles Perkins Centre, University of Sydney, Sydney, NSW, Australia; McMaster University, CANADA

## Abstract

Type 1 diabetes is an autoimmune condition characterised by pancreatic beta cell destruction and absolute insulin deficiency. The strongest predictor of diabetes complications is glycaemic control and achieving HbA1c ≤ 7.0% is the primary management target. However, standard treatment appears to be lacking and adjunctive strategies require consideration. A systematic review was conducted to examine the effect of low-carbohydrate diets on type 1 diabetes management. Four databases were searched from inception until 28 March 2017: MEDLINE; CINAHL; Cochrane Library; and EMBASE. All primary studies containing a methods section (excluding cross-sectional) were included. Reports had to quantitatively measure the effect(s) of a dietary intervention or observed intake over at least two weeks where carbohydrate is below 45% total energy in adults and/or children with type 1 diabetes. The primary outcome was HbA1c and secondary outcomes were severe hypoglycaemia, total daily insulin, BMI, quality of life and mean daily glucose. Seventy-nine full-text articles were assessed for eligibility and nine were included (two randomised controlled trials, four pre-post interventions, two case-series, one case-report). Eight studies reported a mean change in HbA1c with a low-carbohydrate diet. Of these, four reported a non-significant change (P ≥ 0.05) and three reported statistically significant reductions (P < 0.05). Two studies reported severe hypoglycaemia, five reported total insulin, three reported BMI, and one reported blood glucose. Due to the significant heterogeneity of included studies, an overall effect could not be determined. This review presents all available evidence on low-carbohydrate diets for type 1 diabetes and suggests an urgent need for more primary studies.

## Introduction

Type 1 diabetes is an autoimmune condition characterised by the destruction of pancreatic beta cells and absolute insulin deficiency. Affected individuals have impaired glucose metabolism and are prone to chronic complications from hyperglycaemia, and acute complications from hypoglycaemia and ketoacidosis. The standard treatment consists of daily injections of insulin and diet flexibility is encouraged.

The strongest predictor of diabetes complications is glycaemic control and achieving normal glycated haemoglobin (HbA1c ≤ 7.0% or 53 mmol/mol) is considered the primary target in diabetes management [1–4]. However, data from type 1 diabetes registries across nineteen countries in Australasia, Europe and North America (*n* = 324,501) reported that 84% of patients exhibited HbA1c above this target [5]. It appears that current therapies are lacking in effect and adjunctive strategies require consideration.

Low-carbohydrate diets are defined according to the American Diabetes Association (ADA) classifications of less than 130 g/day or 26% total energy intake (TEI) from carbohydrate. Prior to the discovery of insulin, strict low-carbohydrate diets were an accepted method for treatment of diabetes with severe carbohydrate restriction (≤10 g/day) or water-fasting also prescribed until glycosuria was eliminated [6]. More recently, a large observational study of 1020 European outpatients with type 1 diabetes reported that a lower intake of total carbohydrate was associated with lower levels of HbA1c [7].

In type 1 diabetes, blood glucose excursions are a function of the input of glucose from food, mainly carbohydrates (starch and sugars), and insulin from predominantly exogenous sources [8]. By reducing dietary carbohydrate, the error rate in determining the required exogenous insulin amount is reduced and blood glucose fluctuations attenuate [4]. Consequently, less frequent and severe hyper- and hypoglycaemic episodes as well as a reduction in overall insulin requirements should result [9]. Demonstration of these benefits with carbohydrate restriction in type 1 diabetes patients have been recently reported [8, 10].

However, in accordance with the National Health and Medical Research Council recommendations for the management of type 1 diabetes in Australia, patients are advised to consume carbohydrates to the level of 45–65% total energy intake [11–12]. Approaches that promote diet and insulin flexibility, such as Dose Adjustment For Normal Eating (DAFNE), are encouraged by health professionals. However, these approaches rely heavily on carbohydrate counting and insulin dose adjustments. A qualitative review of DAFNE participants highlights however, that many patients chose to severely restrict carbohydrate as they found large amounts of carbohydrate coupled with large insulin doses led to unpredictable blood glucose results [13].

Given the contradiction between recent evidence for carbohydrate restriction in type 1 diabetes management and the NHMRC recommendations, we conducted a systematic review of the literature examining the effect of all low-carbohydrate diets for the management of type 1 diabetes mellitus. We set out to determine whether significant differences in type 1 diabetes management outcomes exist between low-carbohydrate diets and higher-carbohydrate comparators. We also investigated whether primary nutrition studies of low-carbohydrate diets have different levels of effect depending on the degree of carbohydrate restriction.

## Research design and methods

This systematic review was conducted following a registered protocol (http://www.crd.york.ac.uk/PROSPERO/display_record.asp?ID=CRD42017060113) and reported following the Preferred Reporting Items for Systematic Reviews and Meta-Analyses (PRISMA 2009) guidelines (S1 Checklist) [14].

### Data sources and searches

The following databases for health sciences were systematically searched from inception until 28 March 2017: MEDLINE; CINAHL; Cochrane Library; and EMBASE. Our search terms combined the population with the intervention. The medical subject headings used were ‘Diabetes Mellitus, Type 1’, ‘Insulin Dependent Diabetes Mellitus’, ‘Insulin Deficiency’, ‘Diet, Carbohydrate-Restricted’, ‘Ketogenic Diet’, ‘Low Carbohydrate Diet’, ‘Diet, Low Carbohydrate’, and ‘Diet, Ketogenic’. The complete search strategy for Ovid MEDLINE is shown in S1 Table. The search was restricted to human studies published in English. Citations and abstracts of all papers retrieved from these searches were downloaded into Endnote reference management software (Endnote X7.7.1, Thomson Reuters 2016). Reference lists of included studies were also searched.

### Study selection

Two reviewers (JT & KR) independently screened titles and abstracts of all retrieved records for obvious exclusions. The reviewers (JT & KR) then independently assessed the remaining papers based on full text, applying pre-specified eligibility criteria for included studies. Disagreements were resolved by consensus through adjudication with a third independent researcher. Studies included in the review had to be primary research studies of interventions or exposures including controlled trials, cohort-type studies and case-control trials. Case-series and case-reports with a methods section could also be included. In the case of multiple reports from the same study, we used the most complete or recently reported data. The studies had to quantitatively measure the effects of a dietary intervention or observed mean intake below the AMDR for carbohydrate (i.e., <45% total energy as dietary carbohydrate) in adults and/or children with type 1 diabetes that were otherwise healthy. The minimum duration of the intervention or exposure period was two weeks and the study had to adequately report on at least one of the following outcomes; HbA1c, severe hypoglycaemia, total daily insulin dose, body mass index (BMI), quality of life and mean daily blood glucose.

### Data extraction and quality assessment

Data extraction was carried out by JT using a piloted customized data extraction form adapted from the Cochrane Collaboration [15] (S2 Table). For studies investigating different levels of carbohydrate restriction, the lowest reported or prescribed level of dietary carbohydrate intake was considered the intervention and the highest level was considered the comparator. One reviewer (JT) contacted authors for relevant data that were missing from the included papers (S3 Table).

Risk of bias assessments were conducted for methodological quality of each included study using the critical appraisal tool most appropriate for its design. For randomised controlled trials, the Cochrane Collaboration’s Risk of Bias tool for randomised studies was used [16]. This assesses bias as ‘low risk’, ‘high risk’ or ‘unclear risk’ across seven domains. For specificity, we separated blinding of participants and blinding of personnel into two separate domains. For pre-post intervention studies, the National Institute of Health’s quality assessment tool for before-after studies with no control group was used [17]. This tool evaluates potential flaws in study methods or implementation using twelve closed questions. The ratings (yes/no/other) on the different items are then used by reviewers to assess overall risk of bias as ‘good’ (low risk of bias), ‘fair’ (susceptible to some bias) or ‘poor’ (significant risk of bias). For case-series and case-reports, we used the critical appraisal checklists from the Joanna Briggs Institute [18]. These checklists are a series of 8 to 10 closed questions (yes/ no/unclear/not applicable) which help form an overall appraisal for each study assessed. For standardisation, we used this assessment to classify studies as ‘low risk’, ‘high risk’ or ‘unclear risk’ of bias. All risk of bias assessments were performed at the study level. However, when information was specifically related to outcome measures (e.g., ‘blinding of outcome assessment’) judgement was made according to our primary outcome, HbA1c. If HbA1c was not measured, the next reported secondary outcome was used. If a decision could not be reached on bias assessments, an additional investigator made a decision.

The GRADE approach was used to assess the quality of the body of evidence at the outcome level for our primary outcome, HbA1c [19]. This involved consideration of within-study risk of bias, consistency, directness of evidence, precision of effect estimates and risk of publication bias. This approach specifies four levels of quality; ‘high', 'moderate', 'low' and 'very low’.

### Data synthesis and analysis

To summarise the effects of low-carbohydrate diets on type 1 diabetes outcomes in controlled trials, we extracted mean outcome values for the intervention and control groups at baseline and follow-up. For other studies with only an intervention group or for trials where only one participant group was relevant to our study, we extracted mean outcome values for the intervention or observed group at baseline and follow-up. Standard deviations and/or standard errors, sample sizes, follow-up time and published levels of significance (i.e., P-values) were also taken. If no P-value was published and raw outcome data were available, the P-value was calculated using the R Statistical Language (R version 3.4.0, R Foundation for Statistical Computing 2017). Results were considered statistically significant if P < 0.05 and non-significant if P ≥ 0.05. Standard errors were converted to standard deviations by SD = SE x √*n*.

A meta-analysis was not able to be conducted due to obvious heterogeneity and we used text and tabular format to summarise the outcome data of all low-carbohydrate diets. Studies were classified into one of three groups, determined by the dietary carbohydrate amount of the intervention prescription where a g/day or %TEI value was available. Where no specific prescription was available and compliance to the intervention was reported, studies were classified according to the reported dietary intake data of participants. Very Low Carbohydrate Ketogenic Diet (VLCKD) studies are those in which the intervention is less than 50 grams of total carbohydrate per day. True Low Carbohydrate Diet (TLCD) studies are those in which the intervention is 50–130 grams of total carbohydrate per day. False Low Carbohydrate Diet (FLCD) studies are those in which the intervention is below the AMDR for carbohydrate (i.e., <45% total energy) but does not meet the ADA criteria for a low-carbohydrate diet (i.e., <130 grams of total carbohydrate per day or <26% total energy as carbohydrate).

## Results

### Literature search results

**Fig 1:**
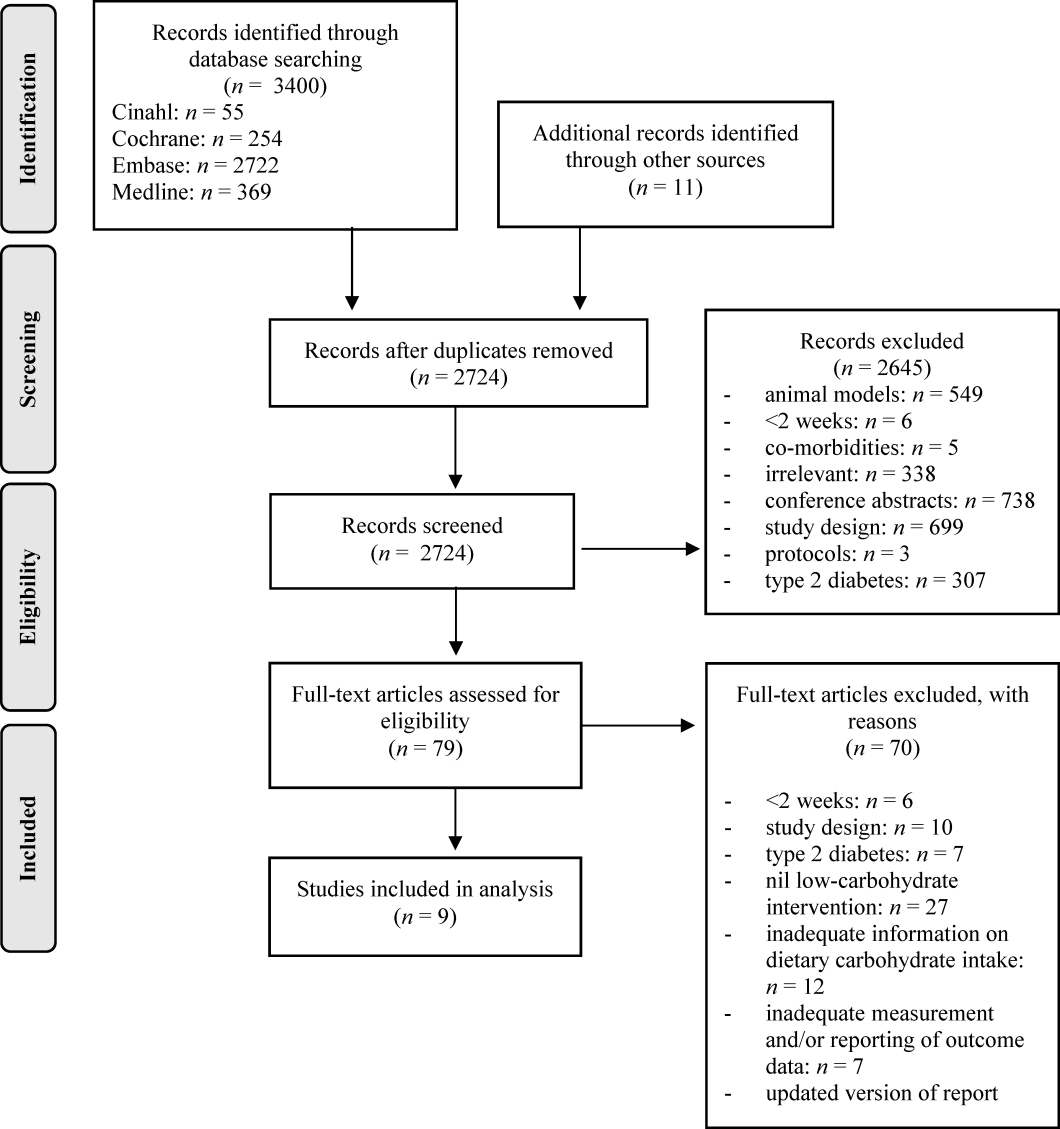
PRISMA flow chart.

The database search identified 2724 possibly relevant studies that were screened by titles and abstracts (Fig 1). A further 2645 records were excluded with 79 full-text articles subsequently assessed for eligibility. Six articles were excluded because the intervention was less than two weeks, 10 studies were excluded because the study design was a review, letter or cross-sectional, seven studies were excluded because no type 1 diabetes mellitus sub-group were analysed, 27 studies were excluded because there was no low-carbohydrate diet intervention, 12 studies were excluded because inadequate dietary data were reported, seven studies were excluded because there were inadequate measurement and/or reporting of outcome data, and one study was excluded because an updated version of the report/data exists. Eleven additional records were identified through searching the reference lists of included studies. A total of nine studies were eligible and included for this review. A full list of excluded studies with reasons is provided (S4 Table).

### Study characteristics

Of the nine included studies, there were two randomised controlled trials [10, 20], four pre-post intervention studies [8, 21–23] two retrospective case-series [24–25], and one case-report [26]. These studies are heterogeneous with respect to study design, sample size, intervention, comparator, outcomes assessed and study quality (Table 1). Year of publication ranged from 1980 to 2016 and the number of participants ranged from 1 to 48. The duration of exposure to a low-carbohydrate diet ranged from two weeks to five years. The two controlled trials compared a low-carbohydrate diet (intervention) to a higher-carbohydrate diet (comparator) using either a crossover [20] or parallel [10] design. All other studies compared a low-carbohydrate diet (intervention) to baseline or usual diet (comparator).

**Table 1:** Characteristics of included studies.

Study Details	Population^a^	Intervention^b^	Comparator^b^	Insulin Protocol^c^	Outcome^d^
**Anderson 1991 [20].** United States. RCT (crossover).	*n* 10. 47 (35–65) y. 18 (1–40) y_D_.	Low-carbohydrate, low-fiber diet. 4 weeks (28 days). Metabolic ward (inpatients). Meals provided. C: 39%, P: 20%, F: 41%. Weight-maintaining.	High-carbohydrate, high-fiber diet. 4 weeks (28 days). Metabolic ward (inpatients). Meals provided. C: 70%, P: 20%, F: 10%.	*Controlled*. Neutral protamine Hagedorn and Regular human insulin subcutaneously before breakfast, lunch and dinner. BG goal: <8.33 mmol/L at 0700, 1100, 1600 & 2200 h daily.	HbA1c
**Bernstein 1980 [26].** United States. Case report.	*n* 1. 45 y. 33 y_D_.	Low-carbohydrate, high-protein diet. 5 years. C: 15% (1 exchange/meal), P: ≥45%, F: ≤40%.	-	*Controlled*. Daily insulin dose (0.4 U/kg body weight) split via three injections. 10 U Ultralente taken in two basal doses. 15 U Regular insulin taken in three preprandial doses. Dosages assessed according to CHO and protein ingested. BG goal: 100 mg/dl (monitored 6x daily)	SH, TDI.
**Chantelau 1982 [21].** Germany. Pre-post intervention (multiple parts).	*n* 10. 26 (18–34) y. 14 (6–24) y_D_.	“Less restricted diabetes diet”–free choice of number, timing and CHO content of meals (as compared to American Diabetes Association diabetes diet). 4–5 months.	-	*Controlled*. Ambulatory CSII therapy. CSII installed 4–5 wk before study started. Insulin dose given 15 min before meals and assessed according to CHO amount ingested and pre-prandial glycemia. BG goal: post-prandial ≤160 mg/dl (monitored 15 min before and 60 min after meals)	HbA1c
**Ireland 1992 [22].** Australia. Pre-post intervention (multi-arm).	*n* 8. 33 (26–43) y. 2–35 y_D_.	Low fat, low carbohydrate diet. 2 weeks. Major meals provided. Breads and cereals ≤100 g/d. Raw lean meat: 1 kg/d. Low-fat/skim dairy ≤ 500 mL/d. Isocaloric (with control).	Self-selected (control) diet (i.e., baseline). 2 weeks. Food not supplied.	*Not controlled*. All subjects followed conventional twice daily insulin regimens (observed). All subjects practiced blood glucose monitoring, performing ~10 tests/wk and appropriate adjustments were made to insulin dose if required.	HbA1c, TDI.
**Knight 2016 [23].** Australia. Pre-post intervention.	*n* 46. 40 (29–51) y. 20 (11–26) y_D_.	DAFNE program–“increased dietary freedom”. 12 months. No dietary prescription of macronutrient intake.	Pre-course (usual diet).	*Not controlled*. Flexible insulin therapy program (DAFNE) with focus on acquisition of patient-based skills in insulin adjustment.	HbA1c, SH.
**Krebs 2016 [10].** New Zealand. RCT (parallel).	*n* 10. 45 y. 22 (8–36) y_D_.	Carbohydrate restricted diet with carbohydrate counting. 12 weeks. C: 50–75 g/d. Carbohydrate counting course (4x 1.5 h sessions), written resources, telephone access to dietitian and diabetes nurse.	Standard diet with carbohydrate counting. 12 weeks. *As per intervention*.	*Controlled*. Within the carbohydrate counting course, education was provided on the action of insulin, insulin to carbohydrate ratios, correction factors and managing sick days. Information was provided on the amount of insulin likely needed to match 50–75 g of carbohydrate per day.	HbA1c, TDI, BMI, BG.
**Nielsen 2012 [8].** Sweden. Pre-post intervention.	*n* 48. 52 y. 24 y_D_.	Carbohydrate restricted diet. 4 years. C: ≤75 g/d (15–20%), P: 30%, F: 50–55%. Group education course (whole day followed by 4 x 3 h sessions over 4 wk). Recipes and sample menus provided.	-	*Controlled*. Patients without insulin pump were switched to Aspart in a pen device that enables delivery of half-units. The insulin treatment consists of two arms: basal and rapid-acting meal insulin.	HbA1c, TDI, BMI.
**O’Neill 2003 [24].** United States. Case-series (retrospective).	*n* 10. 42 (14–60) y. 21 (5–31) months_D_.	Carbohydrate-restricted diet. 18.7 (8–61) months. C: 30 g/d. Snacking prohibited. 3-d clinical evaluation and explanation of program. Phone calls and office visits used to tailor individual regimen of each patient.	-	*Controlled*. All injections <7 units. Patients instructed to wait 5 h between subsequent bolus injections, and no more than 9 h between evening basal dosages and morning basal dosages. For corrections, patients instructed to inject very small dosages (~1/4 unit). BG monitoring ≥4 times daily.	HbA1c, TDI.
**Vernon 2003 [25].** Case-series (retrospective).	*n* 1. 38 y_D_.	Carbohydrate-restricted dietary approach. 3 months. C: (1) ≤20 g/d (2) 5 g added each week until no urinary ketones. Daily multivitamin and omega-3 supplementation.	-	*Controlled*. In diabetics taking more than 10 U/d, the dose was reduced by 50% at the initiation of diet. If BG above 350 mg/dL for more than 3 days after initiation, insulin was increased. BG goal: 150–200 mg/dL.	HbA1c, BMI.

Abbreviations: RCT (randomised controlled trial), y (years), CHO (carbohydrate), C (total daily dietary carbohydrate), P (total daily dietary protein), F (total daily dietary fat), BG (blood glucose), CSII (continuous subcutaneous insulin infusion), DAFNE (Dose Adjustment for Normal Eating), g/d (grams/day), h (hour/s), min (minutes), wk (week/s), y (years), U (units of insulin), mg (milligrams), dL (deciliter).

a: Sample size (*n*) includes participants with Type 1 Diabetes (only) who completed the study in the intervention and/or comparator group specified. Age is given as the mean (to nearest whole year) and range of *n* participants. Diabetes duration (*x*_*D*_) is given as the mean (to nearest whole year) and range of *n* participants.

b: Items include definition (verbatim), duration; main method/s of delivery; macronutrient prescription(s) in g/d and/or percent total energy intake (%) for each macronutrient (P, C, F); and, total energy allowance (verbatim) if indicated in study.

c: *Controlled*–Researchers made an acceptable attempt to control for the effect of insulin (on HbA1c). *Not controlled*–Researchers did not make an acceptable attempt to control for the effect of insulin and/or only observed insulin protocols (i.e., usual methods of participants) were documented.

d: Primary and secondary outcomes of this study that were measured and reported in included study of interest: Haemoglobin A1c (HbA1c), severe hypoglycemia (SH), total daily insulin (TDI), mean daily blood glucose (BG). Quality of life was not measured in any of the nine included studies.

Three studies were classified as FLCD[20, 21, 23], four studies were classified as TLCD [8, 10, 22, 26], and two studies were classified as VLCKD [24–25] (S5 Table). Seven studies [8, 10, 20–21, 24–26] attempted to control for the confounding effect of insulin therapy and two studies [22–23] did not (Table 1).

### All outcomes

Results for all primary and secondary outcomes are presented in Table 2. Effect sizes were not calculated because raw outcome data were not available for all studies and most outcomes were inconsistently reported. In one study [8], outcome data was provided for both all participants (I_A_) and adherent participants only (I_B_) as shown in Table 2. Results for our primary outcome (HbA1c) were available from eight of nine studies reviewed. Results for secondary outcomes of interest were inconsistently reported. Two studies reported the effect of a low-carbohydrate diet on frequency of severe hypoglycaemia [23, 26], five studies reported total daily insulin [8, 10, 22, 24, 26], three studies reported BMI [8, 10, 25], and one study reported mean daily blood glucose [10]. No studies adequately reported change(s) in quality of life. Only results from the most frequently reported outcomes are described below.

**Table 2:** Effect of intervention and comparator diets on type 1 diabetes management outcomes (primary and secondary).

Study ID	I/C^a^	CHO^b^	Class^c^	Follow-up^d^	Pre (units)^e^	Post (units)^e^	Significance^f^	
							Within Group	Between Groups
**Haemoglobin A1c** *(%HbA1c (mmol/mol))*								
Anderson 1991	I	221 ± 35 (~39%)	FLCD	4 weeks (*n* 8)	9.0 ± 0.5 (75 ± 5.5)	8.1 ± 0.4 (65 ± 4.4)	NS	NS
	C	363 ± 76 (~68%)	-	4 weeks (*n* 8)	9.0 ± 0.5 (75 ± 5.5)	8.0 ± 0.3 (64 ± 3.3)	NS	
Chantelau 1982	I	156 ± 46 (34 ± 5%)	FLCD	4–6 months (*n* 10)	9.7 ± 1.9 (83 ± 20.8)	7.3 ± 0.5 (56 ± 5.5)	P < 0.0025	-
Ireland 1992	I	~87 (22 ± 6%)	TLCD	2 weeks (*n* 8)	11.1 ± 1.7 (98 ± 18.6)	11.6 ± 2.3 (103 ± 25.1)	NS	-
Knight 2016	I	162 (143–204) (42 ± 7%)	FLCD	12 months (*n* 46)	7.9 ± 1.2 (63 ± 13.1)	7.9 ± 1.6 (63 ± 17.5)	NS	-
Krebs 2016	I	103 ± 22 (~30%)	TLCD	12 weeks (*n* 5)	7.9 ± 0.9 (63 ± 9.8)	7.2 ± 0.4 (55 ± 4.4)	NS	NS
	C	203 ± 92 (~44%)	-	12 weeks (*n* 5)	7.4 ± 0.9 (57 ± 9.8)	7.4 ± 0.9 (57 ± 9.8)	NS	
Nielsen 2012	I_A_	≤75 (15–20%)	TLCD	4 years (*n* 48)	7.6 ± 1.0 (60 ± 10.9)	6.9 ± 1.0 (52 ± 10.9)	P < 0.001	-
	I_B_	-	-	4 years (*n* 23)	7.7 ± 1.0 (61 ± 10.9)	6.4 ± 0.8 (46 ± 8.7)	P < 0.001	-
O’Neill 2003	I	30	VLCKD	18 months (*n* 8)	6.8 ± 1.1 (51 ± 12.0)	5.5 ± 0.8 (37 ± 8.7)	P = 0.003	-
Vernon 2003	I	20–50	VLCKD	3 months (*n* 1)	16.8 (160)	5.3 (34)	NA	-
**Severe Hypoglycemia** *(episodes/year)*								
Bernstein 1980	I	15%	TLCD	5 years (*n* 1)	730.0^g^	12.0	NA	-
Knight 2016	I	162 (143–204) (42 ± 7%)	FLCD	12 months (*n* 46)	3.7 ± 15.7	0.2 ± 1.1	P = 0.014	-
**Total Daily Insulin** *(units/day)*								
Bernstein 1980	I	15%	TLCD	5 years (*n* 1)	80.0	25.0	NA	-
Ireland 1992	I	~87 (22 ± 2%)	TLCD	2 weeks (*n* 8)	41.1 ± 3.5	35.3 ± 4.1	P < 0.05	-
Krebs 2016	I	103 ± 22 (~30%)	TLCD	12 weeks (*n* 5)	66.4 ± 25.3	44.2 ± 16.5	P < 0.05	P < 0.05
	C	203 ± 92 (~44%)	-	12 weeks (*n* 5)	40.6 ± 7.8	44.8 ± 12.4	NS	
Nielsen 2012	I	≤75 (15–20%)	TLCD	1 year (*n* 36)	42.6 ± 10.3	31.6 ± 8.5	NA	-
O’Neill 2003	I	30	VLCKD	8–61 months (*n* 10)	47.0	30.0	NA	-
**Body Mass Index** *(kg/m*^*2*^*)*								
Krebs 2016	I	103 ± 22 (~30%)	TLCD	12 weeks (*n* 5)	27.5 ± 2.2	25.8 ± 1.0	NS	NS
	C	203 ± 92 (~44%)	-	12 weeks (*n* 5)	27.7 ± 6.2	27.6 ± 6.1	NS	
Nielsen 2012	I	≤75 (15–20%)	TLCD	4 years (*n* 48)	25.9 ± 3.5	25.7 ± 3.8	NS	-
Vernon 2003	I	20–50	VLCKD	3 months (*n* 1)	20.5	23.3	NA	-
**Mean Blood Glucose** *(mmol/L)*								
Krebs 2016	I	103 ± 22 (~30%)	TLCD	12 weeks (*n* 5)	10.2 ± 2.3	8.9 ± 0.8	NS	NS
	C	203 ± 92 (~44%)	**-**	12 weeks (*n* 5)	9.3 ± 1.9	10.1 ± 2.9	NS	

a: Intervention group (I), comparator group (C), Intervention group—all participants (I_A_), Intervention group–adherent participants only (I_B_).

b: CHO (dietary carbohydrate) is actual dietary intake of participants reported in grams/day as $\bar{x}$ ± SD or $\bar{x}$ (range) (to nearest whole unit) and/or as percent total energy (%), where available. ~ indicates that value was calculated using Atwater factors and not taken from study (i.e., not reported). Reported as intervention prescription where no actual dietary data available.

c: Intervention classification: false low-carbohydrate diet (FLCD) (>130 g/d); true low-carbohydrate diet (TLCD) (50–130 g/d); very low-carbohydrate ketogenic diet (VLCKD) (<50 g/d).

d: Follow-up coincides with duration of exposure to intervention for all studies. Sample size (*n*) of intervention group and/or comparator in parentheses.

e: Outcome values are presented as $\bar{x}$ ± SD (standard deviations) (to 1 decimal place) in units expressed as per outcome. HbA1c also presented as $\bar{x}$ in mmol/mol (to nearest whole unit) ± SD in mmol/mol (to 1 decimal place) in parentheses.

f: Level of significance (P-value) given as reported in study, or calculated to 3 decimal places (if raw data were available). Not-significant (NS) assigned to changes where P ≥ 0.05 or if indicated as NS in study. Not applicable (NA) assigned where no standard deviations were reported in study, sample size is 1 and/or raw data were not available for calculations.

g: Frequency of severe hypoglycemia was reported as an average of 2 episodes daily. This was converted to 730 via simple calculation (2 x 365) and may not be an accurate representation of a full year.

### HbA1c

Eight studies investigated the effect of a low-carbohydrate diet on glycaemic control using HbA1c [8, 10, 20–24, 26] (Table 2). One study [26] reported a follow-up value for HbA1c but did not provide baseline data so was not included for this outcome. Four studies [10, 20, 22–23] reported non-significant changes in HbA1c with a low-carbohydrate diet and three studies [8, 21, 24] reported statistically significant reductions (P < 0.05). Of the two studies that compared a low-carbohydrate diet to a higher-carbohydrate diet [10, 20], neither showed a significant difference between groups at follow-up.

### Total daily insulin

Of the five studies that reported daily insulin usage [8, 10, 22, 24, 26], two TLCD studies [10, 22] demonstrated statistically significant reductions in total daily insulin within carbohydrate restriction groups (P < 0.05) (Table 2) with one study [10] also reporting a statistically significant difference between the low-carbohydrate group and high-carbohydrate comparator (P < 0.05). Levels of significance could not be calculated or obtained in three studies due to inadequate sample size [26] and lack of raw participant data [8, 24].

### Risk of bias assessments

Full assessments of the two RCTs reviewed with support for judgements are included in S6–S8 Tables. Risk of bias in one [20] was rated ‘low’ in four domains and ‘unclear’ in four domains. The other controlled trial [10] was rated ‘low’ in seven domains and ‘unclear’ in one domain. Of the pre-post intervention studies (S9–S13 Tables), two were rated as ‘fair’ quality [8, 21] and two were rated as ‘poor’[22–23]. The poor-quality studies did not attempt to control for the confounding influence of insulin therapy on HbA1c. One case-series [24] had an overall risk of bias of ‘high’ and the other case-series [25] had a ‘low’ risk of bias (S14 Table). Both reports had clear criteria for inclusion, valid methods for identification of type 1 diabetes, clear outcome results of cases and appropriate statistical analyses. The case-report [26] had an overall appraisal of ‘low’ risk of bias (S15 Table).

### GRADE assessment

The quality of evidence could not be upgraded from the established level of confidence because the statistical analyses required for such categories (i.e., large magnitude of effect, dose-response gradient, effect of plausible residual confounding) were not performed. The five categories used for downgrading the quality of evidence were assessed and included in Table 3. Categories were assigned a rating of zero if the appropriate statistical analyses required to confidently downgrade the evidence based on the criteria were not able to be performed, or if it could be appropriately justified that the evidence should not be downgraded for that category.

**Table 3:** Summary of findings table (GRADE) for primary outcome (HbA1c).

Outcome	No. of participants (studies). *Follow up*.	Category	Rating with Reasoning	Quality of the evidence (GRADE)^a^
HbA1c	139 (8 studies) [8, 10, 20–25]. *2 weeks– 4 years*.	*Study design*	*Established level of confidence (study design) was set to ‘low’ due to the inclusion of 2 randomised and 6 non-randomised studies*.	⊕OOO *Very low*
		Risk of bias	Three studies rated were rated with unsatisfactory judgements (i.e., ‘poor’ quality, ‘high’ risk of bias) (-1).	
		Consistency	Consistency could not be statistically assessed as no meta-analysis was performed (0).	
		Directness	The evidence is highly applicable to our relevant question (PICO) as HbA1c is the primary outcome for diabetes management (0).	
		Precision	Precision could not be statistically assessed as no meta-analysis was performed (0).	
		Publication bias	It is unlikely that additional studies have been conducted on this specific topic due to the perceived risk involved in reducing carbohydrate below recommended levels in patients with type 1 diabetes. We were unable to create a funnel plot to support this judgement as this requires at least 10 studies and there are only 8 studies for this outcome (0).	

Abbreviations: PICO (population, intervention, comparator, outcome). Population: Adults with type 1 diabetes that are otherwise healthy. Intervention: Low-carbohydrate diet (i.e., <45% total energy intake as carbohydrate). Comparator: Higher-carbohydrate diet (i.e., observed baseline diet or separate intervention).

a: Available ratings include ‘very low’, ‘low’, ‘moderate’, ‘high’.

## Discussion

The present systematic review is the first of its kind to present all available evidence on low-carbohydrate diets for the management of type 1 diabetes. Due to the significant heterogeneity of included studies, we were unable to conclusively determine whether significant differences in type 1 diabetes outcomes exist between low-carbohydrate diets and higher-carbohydrate comparators. We were also unable to determine whether primary nutrition studies of low-carbohydrate diets have different levels of effect depending on the degree of carbohydrate restriction. However, for all studies reporting a significant change in HbA1c, the direction of change was similar with low-carbohydrate interventions or observed intakes. This review highlights a limited body of evidence and suggests the need for more high-quality prospective trials examining the effect of low-carbohydrate diets in the management of type 1 diabetes.

### Clinical significance of results

The United Kingdom Prospective Diabetes Study highlighted the importance of lowering HbA1c to reduce the risk of micro and macrovascular complications in patients with diabetes [1]. Further, Juutilainen and colleagues (2008) reported that in type 1 diabetes patients, a 1% rise in HbA1c increased individual risk of cardiovascular mortality by 52.5% [27]. Cardiovascular diseases are the main cause of morbidity and mortality in diabetes, affecting about 55% of patients, compared to 2–4% of people without diabetes [28]. Our review identified statistically significant improvements in glycaemic control in 3 of 8 studies (*n* > 1) that reported a change in mean HbA1c with a low-carbohydrate diet [8, 21, 24]. In addition, the importance of non-significant changes in HbA1c (i.e., maintaining glucose levels) reported in the other 5 studies [10, 20, 22, 23] is worth noting considering the natural progression for diabetes is toward poorer glycaemic control, preventing HbA1c from rising could be considered a successful outcome.

In this review, all five studies reporting total daily insulin showed clinically significant reductions with a low-carbohydrate diet [8, 10, 22, 24, 26]. The excessive use of insulin that is often required to achieve glycaemic control in type 1 diabetes increases susceptibility to severe hypoglycaemia and may lead to some measure of hyperinsulinemia [29]. Hyperinsulinemia is associated with; excessive weight gain [30], development of the metabolic syndrome [31], inflammation and atherosclerosis [32], Alzheimer’s Disease [33] and cancer [34]. Findings of the present review suggest that low-carbohydrate intakes may assist in reducing or preventing hyperinsulinemia in type 1 diabetes by decreasing the absolute amount of insulin required for tight glycaemic control.

Insulin therapy is also a major confounder in all studies attempting to examine the effect of a lifestyle intervention on HbA1c in type 1 diabetes. The process of randomisation attempts to control for the potential differences in insulin therapy protocols within a study population. However, in non-randomised studies, controlling for insulin can be difficult. In the current review, insulin protocols of participants are presented in Table 1 as they were reported in the study. We classed these as ‘controlled’ if an attempt was made to standardise the insulin protocol of participants or ‘not controlled’ if researchers did not intervene or flexible protocols were promoted. These classifications contributed to the overall risk of bias, and studies that did not control for insulin [22–23] could not receive low-level judgements (‘low’ risk of bias or ‘good’ and ‘fair’ quality).

### Strengths of present review

A major strength of the present review is the inclusion of a wide variety of study designs to capture all the available evidence and effectively serve as a library of all published data on low-carbohydrate diets in type 1 diabetes management to date. Standard systematic reviews that set out to evaluate the effect(s) of an intervention tend to exclude evidence based on factors such as small sample size (e.g., *n* = 1), lack of data (e.g., reported dietary intake) and study design (e.g., non-randomised). This approach is useful in informing public policy and national dietary guidelines. However, this method of review fails to collect data that could be particularly useful in areas of specialised practice where conclusive evidence is limited.

To assist with the interpretation of this review, we performed rigorous risk of bias and quality assessments of all included papers at the study and outcome levels. Use of the GRADE criteria addressed the inherent bias that could have been introduced with the inclusion of low levels of evidence, such as case-reports [35], which was not necessarily identified in the study-level risk of bias assessments (Table 3). Multiple study-level risk of bias assessments were performed when the review authors considered the original appraisal tool to be potentially insufficient in assessing the quality of a paper. For example, two studies were experimental in design, yet the low-carbohydrate diets we assigned as interventions were more accurately, observed outcomes (i.e., exposures) [21,23]. The original appraisal tool applied is specific to intervention studies [17], and the additional appraisal tool is specific to studies of exposures. The Cochrane Collaboration’s tool for assessing risk of bias in non-randomised studies of exposures (ROBINS-E) [36] was used and though ROBINS-E is currently under development, its application is cited in existing review protocols [37]. Results were considered consistent with the original assessments (S16 and S17 Tables).

### Limitations of review

Several limitations of our study should be acknowledged. Our search only focused on four online databases. As a result, studies from other relevant journals or grey literature may have been missed. The decision to include studies ‘from inception’ meant that many corresponding authors could not be contacted to retrieve data. For the same reason, methods of outcome measurements may not have been as accurate or reliable as methods used in more recent studies. In addition, we were unable to calculate meaningful effect sizes or conduct a meta-analyses due to the inclusion of multiple study designs, small sample sizes and reports with inadequate reporting of raw participant data and/or standard deviations. It should also be acknowledged that HbA1c is generally considered a three-month average and studies with a follow-up of less than three months may not have been sufficient to detect a true effect. Nevertheless, inadequate follow-up periods were addressed in the risk of bias assessments.

Some limitations also arose from the inclusion of studies with missing or inadequate reported dietary data of participants. A major potential flaw being that the current review did not explicitly exclude studies where dietary carbohydrate was increased from a very low baseline intake to a level that remained below 45% total energy at follow-up. However, the studies without baseline dietary data included in this review provided enough information to confidently assume that carbohydrate was decreased during the intervention or observation period. Chantelau et. al. (1982) [21] described the diet of participants as it were prior to the study in the text of the report, while three studies [8, 24, 25] explicitly identified the intervention as a “carbohydrate-restricted” diet (S5 Table).

In addition, compliance to the intervention could only be assessed in three studies [8, 10, 20] where both a rigid carbohydrate prescription and adequate reported dietary data of participants were available. Anderson et al. (1991) [20] exhibited excellent compliance among participants, while Krebs et al. (2016) [10] describes a population that exceeded the carbohydrate prescription by more than 20% (+28 g/day) and still fit the classification of the intended intervention. Nielsen et al. (2012) [8] presented results separately depending on the level of adherence to the intervention of participants, yet no reported dietary data was provided.

### Recommendations for further research

With such limited data available for this review, more high-quality studies are necessary to further inform practitioners of patients with type 1 diabetes on the effect(s) of reducing dietary carbohydrate. Data presented here suggests a particular focus on TLCD and VLCKD designed interventions may provide positive steps forward for reductions in HbA1c. Future research should more adequately consider the potential consequences of interventions that aim to reduce HbA1c in this population. Interventions should only be considered effective if HbA1c can be reduced toward target levels without increasing severe hypoglycaemic episodes, total daily insulin, BMI (≥25 kg/m^2^), mean blood glucose, and/or negatively affecting quality of life. Therefore, outcomes of this review should form the minimum set of outcomes that are reported in future type 1 diabetes research. None of the studies in this review measured all six outcomes and only four studies [8, 10, 22, 24] reported complete measurements for both HbA1c and insulin dose.

Another important consideration for future studies is whether to substitute carbohydrate with fat or protein. Vernon et al. (2003) [25] and O’Neill et al. (2003) [24] specifically substituted carbohydrate with fat, while Bernstein (1980) [26] and Ireland et al. (1992) [22] increased protein intake. Nielsen et al. (2012) [8] appeared to increase both fat and protein in relative proportions. This review is unable to draw any conclusion for potential differences in effect and more primary studies are necessary.

### Conclusion

Type 1 diabetes is a chronic disease with severe complications for its mismanagement. To strengthen patient-centered care and improve individual capacity for problem solving and self-management, health professionals should be equipped with the appropriate evidence base to present multiple management strategies to their patients. Dietary strategies can serve as effective adjuncts to pharmaceutical therapy in the treatment of various metabolic diseases.

This systematic review presents all available evidence for low-carbohydrate diets in the management of type 1 diabetes mellitus. The existing body of evidence is limited and more primary studies evaluating the short and long-term effects of low-carbohydrate diets on type 1 diabetes management outcomes are necessary to support its use in practice.

## Supporting information

S1 Checklist PRISMA checklist. (PDF)

S1 Table Search strategy for OVID Medline. (PDF)

S2 Table Custom data extraction form used for included studies. (PDF)

S3 Table Researcher notes from data extraction. (PDF)

S4 Table Excluded studies (from full-text screen) with reasons for exclusion. (PDF)

S5 Table Reported dietary intake data of participants in intervention and comparator Groups of included studies. (PDF)

S6 Table Summary of risk of bias assessments for included randomised controlled trials using the Cochrane Collaboration’s Risk of Bias for randomised controlled trials assessment tool. (PDF)

S7 Table Risk of bias assessment for Anderson et al. (1991) (20) using the Cochrane Collaboration’s Risk of Bias for randomised controlled trials assessment tool. (PDF)

S8 Table Risk of bias assessment for Krebs et al. (2016) (10) using the Cochrane Collaboration’s Risk of Bias for randomised controlled trials assessment tool. (PDF)

S9 Table Summary of quality assessments using the National Institute of Health’s quality assessment tool for pre-post intervention studies with no control group. (PDF)

S10 Table Quality assessment for Chantelau et al. (1982) (21) using The National Institute of Health’s quality assessment tool for pre-post intervention studies with no control group. (PDF)

S11 Table Quality assessment for Ireland et al. (1992) (22) using The National Institute of Health’s quality assessment tool for pre-post intervention studies with no control group. (PDF)

S12 Table Quality assessment for Knight et al. (2016) (23) using The National Institute of Health’s quality assessment tool for pre-post intervention studies with no control group. (PDF)

S13 Table Quality assessment for Nielsen et al. (2012) (8) using The National Institute of Health’s quality assessment tool for pre-post intervention studies with no control group. (PDF)

S14 Table Risk of bias assessment for case-series using Joanna Brigg’s critical appraisal tool for case-series. (PDF)

S15 Table Risk of bias assessment for Bernstein (1980) (26) using Joanna Brigg’s critical appraisal tool for case-reports. (PDF)

S16 Table Risk of bias assessment for Chantelau et al. (1982) (21) using ROBINS-E^a^. (PDF)

S17 Table Risk of bias assessment for Knight et al. (2016) (23) using ROBINS-E^a^. (PDF)
