# Supplementary material for: Low-carbohydrate diets for type 1 diabetes mellitus: A systematic review
Source: PLoS One. 2018 Mar 29;13(3):e0194987. doi: 10.1371/journal.pone.0194987 (PMC5875783; doi:10.1371/journal.pone.0194987)
Supplement: S1 Table — (PDF) [file pone.0194987.s002.pdf]

S1 Table. Search Strategy for OVID Medline

	Concept(s): Type 1 diabetes mellitus, low-carbohydrate diet
1	Diabetes Mellitus, Type 1/
2	(Diabet* adj3 (type 1? or type I? or T1? or juvenile or insulin dependent or autoimmune or sudden onset)).mp.
3	(absolute adj3 insulin adj3 deficien*).mp.
4	(insulin adj3 therap*).mp.
5	(insulin adj3 inject*).mp.
6	(T1D or T1DM or IDDM or IDD).mp.
7	or/1-6
8	((carb or carbs or carbohydrate*) adj3 (reduc* or restrict* or low* or limit* or deficien*)).mp.
9	((ketone or ketosis or ketogenic or ketotic) adj3 (produc* or diet* or nutrition*)).mp.
10	((diet or protocol) adj3 (CSIRO or south beach or atkins or protein power or dukan or LCHF or zero carb or sugar busters or bernstein* or paleo* or zone)).mp.
11	(high* adj3 fat adj3 diet*).mp.
12	Diet, Carbohydrate-Restricted/
13	Ketogenic Diet/
14	8 or 9 or 10 or 11 or 12 or 13
15	7 and 14
16	limit 15 to humans
