# Supplementary material for: Low-carbohydrate diets for type 1 diabetes mellitus: A systematic review
Source: PLoS One. 2018 Mar 29;13(3):e0194987. doi: 10.1371/journal.pone.0194987 (PMC5875783; doi:10.1371/journal.pone.0194987)
Supplement: S2 Table — (PDF) [file pone.0194987.s003.pdf]

S2 Table: Custom data extraction form used for included studies

**Study ID:**		
**Coder initials:**		
**Date started:**		
**Date completed:**		
**Title of the paper:**		
**Year of publication:**		
**Journal:**		
**Location (country):**		
**Study design**		
1. Randomised controlled trial		
2. Controlled trial / pseudo-randomised / quasi-experimental		
3. Cohort		
4. Clinical audit		
5. Case-control		
6. Case series (retrospective chart review)		
7. Case report		
8. Other design (specify)		
**Population description:**		
**Sample size:**		
**Sex of participants**		
n (males)		
n (females)		
**Age of participants**		
Mean		
Range		
**Duration of participation:**		
**Follow-up period:**		
**Primary aim of study (verbatim):**		
**Intervention / level of exposure**	**Prescription**	**Reported**
Number of participants		
Duration of treatment		
How did the study define the intervention?		
How did the study deliver the intervention?		
Daily intake of total dietary CHO (grams)		
Daily intake of total dietary CHO (% total energy)		
Daily intake of dietary protein (grams)		
Daily intake of dietary protein (% total energy)		
Total daily energy intake	1. Replete<br>2. Restricted	NA
Total daily energy intake (mean) (kcal and kJ)		
Other treatment (additional to diet)		
Classification of intended intervention	1. VLCKD<br>2. TLCD<br>3. FLCD	1. VLCKD<br>2. TLCD<br>3. FLCD
**Compliance to intervention**		
1. Yes (reported CHO within 20% of intended CHO)		
2. No (reported CHO exceeds intended CHO >20%)		
**Type of comparison**		
1. Low-carbohydrate diet vs. low-carbohydrate diet (different doses)		

S2 Table: Custom data extraction form used for included studies

2. Low-carbohydrate diet vs. high-carbohydrate diet		
3. Low-carbohydrate diet vs. control/usual diet		
4. Low-carbohydrate diet vs. other dietary intervention		
5. Low-carbohydrate diet vs. baseline diet (pre-post)		
6. Other		
**Comparator**	**Prescription**	**Reported**
Number of participants		
Duration of treatment		
How did the study define the comparator?		
How did the study deliver the comparator?		
Daily intake of total dietary CHO (grams)		
Daily intake of total dietary CHO (% total energy)		
Daily intake of dietary protein (grams)		
Daily intake of dietary protein (% total energy)		
Total daily energy intake	1. Replete<br>2. Restricted	NA
Total daily energy intake (mean) (kcal and kJ)		
Other treatment (additional to diet)		
**Outcome 1. HbA1c**		
Direction of desirability		
Is outcome tool validated?		
Unit/s reported in study		
Time points measured		
No. of participants		
Intervention	*Absolute Change*	
Baseline value		
Follow-up value		
Effect size (change from baseline)		
Level of significance (P-value or CI)		
Adjusted or unadjusted		
Comparator	*Absolute Change*	
Baseline value		
Follow-up value		
Effect size (change from baseline)		
Level of significance (P-value or CI)		
Adjusted or unadjusted		
Between Groups	*Difference*	
Effect size (difference between groups)		
Effect size (%) (if different units)		
Level of significance (P-value or CI)		
Adjusted or unadjusted		
Direction of result	1. Favours intervention<br>2. Favours comparator	
Other details		
**Outcome 2. Severe hypoglycaemia**		
Direction of desirability		
Is outcome tool validated?		
Unit/s reported in study		
Time points measured		

S2 Table: Custom data extraction form used for included studies

No. of participants	
Intervention	*Absolute Change*
Baseline value	
Follow-up value	
Effect size (change from baseline)	
Level of significance (P-value or CI)	
Adjusted or unadjusted	
Comparator	*Absolute Change*
Baseline value	
Follow-up value	
Effect size (change from baseline)	
Level of significance (P-value or CI)	
Adjusted or unadjusted	
Between Groups	*Difference*
Effect size (difference between groups)	
Effect size (frequency/week)	
Level of significance (P-value or CI)	
Adjusted or unadjusted	
Direction of result	1. Favours intervention
	2. Favours comparator
Other details	
**Outcome 3. Total Daily Insulin**	
Direction of desirability	
Is outcome tool validated?	
Unit/s reported in study	
Time points measured	
No. of participants	
Intervention	*Absolute Change*
Baseline value	
Follow-up value	
Effect size (change from baseline)	
Level of significance (P-value or CI)	
Adjusted or unadjusted	
Comparator	*Absolute Change*
Baseline value	
Follow-up value	
Effect size (change from baseline)	
Level of significance (P-value or CI)	
Adjusted or unadjusted	
Between Groups	*Difference*
Effect size (difference between groups)	
Effect size (units/day)	
Level of significance (P-value or CI)	
Adjusted or unadjusted	
Direction of result	1. Favours intervention
	2. Favours comparator
Other details	
**Outcome 4. BMI**	
Direction of desirability	

S2 Table: Custom data extraction form used for included studies

Is outcome tool validated?	
Unit/s reported in study	
Time points measured	
No. of participants	
Intervention	*Absolute Change*
Baseline value	
Follow-up value	
Effect size (change from baseline)	
Level of significance (P-value or CI)	
Adjusted or unadjusted	
Comparator	*Absolute Change*
Baseline value	
Follow-up value	
Effect size (change from baseline)	
Level of significance (P-value or CI)	
Adjusted or unadjusted	
Between Groups	*Difference*
Effect size (difference between groups)	
Effect size (kg/m^2)	
Level of significance (P-value or CI)	
Adjusted or unadjusted	
Direction of result	1. Favours intervention
	2. Favours comparator
Other details	
**Outcome 5. Quality of life**	
Direction of desirability	
Is outcome tool validated?	
Unit/s reported in study	
Time points measured	
No. of participants	
Intervention	*Absolute Change*
Baseline value	
Follow-up value	
Effect size (change from baseline)	
Level of significance (P-value or CI)	
Adjusted or unadjusted	
Comparator	*Absolute Change*
Baseline value	
Follow-up value	
Effect size (change from baseline)	
Level of significance (P-value or CI)	
Adjusted or unadjusted	
Between Groups	*Difference*
Effect size (difference between groups)	
Effect size (scale value)	
Level of significance (P-value or CI)	
Adjusted or unadjusted	
Direction of result	1. Favours intervention
	2. Favours comparator

S2 Table: Custom data extraction form used for included studies

Other details	
**Outcome 6. Mean Daily Blood Glucose**	
Direction of desirability	
Is outcome tool validated?	
Unit/s reported in study	
Time points measured	
No. of participants	
Intervention	*Absolute Change*
Baseline value	
Follow-up value	
Effect size (change from baseline)	
Level of significance (P-value or CI)	
Adjusted or unadjusted	
Comparator	*Absolute Change*
Baseline value	
Follow-up value	
Effect size (change from baseline)	
Level of significance (P-value or CI)	
Adjusted or unadjusted	
Between Groups	*Difference*
Effect size (difference between groups)	
Effect size (mmol/L)	
Level of significance (p-value or CI)	
Adjusted or unadjusted	
Direction of result	1. Favours intervention
	2. Favours comparator
Other details	
**Sponsorship / funding (verbatim):**	
**Author conflicts of interest:**	

Abbreviations: CHO (dietary carbohydrate), FLCD (false low-carbohydrate diet), TLCD (true low-carbohydrate diet), VLCKD (very low-carbohydrate ketogenic diet), kcal (kilocalorie), kJ (kilojoule), CI (confidence interval).
