# Supplementary material for: Low-carbohydrate diets for type 1 diabetes mellitus: A systematic review
Source: PLoS One. 2018 Mar 29;13(3):e0194987. doi: 10.1371/journal.pone.0194987 (PMC5875783; doi:10.1371/journal.pone.0194987)
Supplement: S3 Table — (PDF) [file pone.0194987.s004.pdf]

S3 Table: Researcher Notes from Data Extraction

Study ID	Notes
Anderson 1991 [20]	Cannot use insulin data as these results were measured (a) from an artificial pancreas, and (b) over 24 hours of consuming 'usual test diet' meals (i.e., not experimental diet meals).
Bernstein 1980 [26]	Cannot use post-values for mean blood glucose or HbA1c because study does not report any initial values for these outcomes.
Chantelau 1982 [21]	Cannot use post-values for mean blood glucose because study does not report any initial values for this outcome. Cannot use data for hypoglycemic events because the follow-up period for this outcome did not coincide with the length of our intervention of interest.
Ireland 1992 [22]	The low-fat, low-carbohydrate diet was used as the intervention because the carbohydrate intake of participants in this group was the lowest (i.e., compared to the high-fat, high-carbohydrate diet). Cannot use data on fasting plasma glucose as these results are not comparable to daily averages (i.e., we only took daily means of pre- and postprandial measurements, or values from a continuous glucose monitoring system)
Knight 2016 [23]	Author contacted (11/05/2017) for mean pre/post values for severe hypoglycemic events and HbA1c.
Krebs 2016 [10]	Cannot use data for quality of life as only a P-value for difference between groups at follow-up was reported (i.e., no mean values of either group at baseline or follow-up were reported).
Nielsen 2012 [8]	Author contacted (04/05/2017) for actual dietary intake data of participants, yet no such data was measured or recorded.
O' Neill 2003 [24]	Author contacted (11/05/2017) for actual dietary intake data of participants.
Vernon 2003 [25]	Author contacted (11/05/2017) for actual dietary intake data and mean pre/post values for total daily insulin of participants.
