# Supplementary material for: Low-carbohydrate diets for type 1 diabetes mellitus: A systematic review
Source: PLoS One. 2018 Mar 29;13(3):e0194987. doi: 10.1371/journal.pone.0194987 (PMC5875783; doi:10.1371/journal.pone.0194987)
Supplement: S4 Table — (PDF) [file pone.0194987.s005.pdf]

S4 Table: Excluded Studies (from full-text screen) with Reasons for Exclusion

Reason for exclusion	No. of studies (*n*)	Excluded studies (study ID)		
No low-carbohydrate intervention, either;<br>• The intervention is within or exceeds the Acceptable Macronutrient Distribution Range for carbohydrate (i.e., ≥ 45% total energy).<br>• There is no significant decrease in carbohydrate intake within intervention group<br>• There is no significant difference in carbohydrate prescription (or actual intake, if observational) to comparator.	*n* 27	Ahola 2012<br>Calle-Pascual 1988<br>Chantelau 1987<br>Chiarelli 1989<br>Delahanty 2009<br>Donaghue 2000<br>Frost 1986<br>Georgopoulos 1998<br>Hollenbeck 1985	Hollenbeck 1985<br>Hollenbeck 1983<br>Ireland 1992<br>Jaacks 2015<br>Kalk 1992<br>Lindsay 1984<br>Lorini 1990<br>Marigliano 2013<br>Marquard 2011	McCulloch 1985<br>Pais 2010<br>Peterson 1986<br>Sarnblad 2005<br>Soedamah-Muthu 2013<br>Strychar 2003<br>Strychar 2009<br>Taskinen 1983<br>Webb 1984
Inadequate information on dietary carbohydrate. Minimum requirement:<br>• Prescription of intervention; and/or<br>• Actual mean intake of participants during intervention/at follow-up	*n* 12	Barnett 1985<br>Burani 2006<br>Cundiff 2002<br>Devitt 2004	Goksen 2014<br>GSEDNu 2006<br>Nansel 2016<br>Rossi 2010	Rossi 2009<br>Salgado 1996<br>Schoenaker 2012<br>Toeller 1999
The duration of the intervention is less than 2 weeks.	*n* 6	Anderson 1979<br>De Loach 2009	Lassenius 2014<br>Ranjan 2017	Riccardi 1984<br>Uthoff 2010
No type 1 diabetes subgroup analysed.	*n* 7	Brunzell 1974<br>Cardot 1997<br>Gallagher 1987	Gardner 2010<br>Kiehm 1976	Stone 1963<br>Ziaee 2012
No primary and/or secondary outcomes are measured, and/or outcome data is inadequate (e.g., no baseline outcome measures reported)	*n* 7	Cresswell 2015<br>Papadaki 2008<br>Rabasa-Lhoret 1999	Sherwin 1981<br>Shimakawa 1993	Simpson 1981<br>Simpson 1979
Study design:<br>• Review (*n* 7);<br>• Letter (*n* 1);<br>• Cross-sectional (*n* 2)	*n* 10	Buyken 2000<br>D'Arrigo 2007<br>Daly 2002<br>Hockaday 1985	Lasserson 2012<br>Manzano 1997<br>Miles 2008	Morrison 2005<br>Rossiter 2000<br>Wylie-Rosett 2014
There is an updated version of the report/data.	*n* 1	Nielsen 2005		
