# Supplementary material for: Low-carbohydrate diets for type 1 diabetes mellitus: A systematic review
Source: PLoS One. 2018 Mar 29;13(3):e0194987. doi: 10.1371/journal.pone.0194987 (PMC5875783; doi:10.1371/journal.pone.0194987)
Supplement: S5 Table — (PDF) [file pone.0194987.s006.pdf]

S5 Table: Reported Dietary Intake Data of Participants in Intervention and Comparator Groups of Included Studies

Study ID	Class[a]	Context/setting	Intervention[b]	Comparator[b]
Anderson 1991 [20]	FLCD	Inpatients. Control (usual diet) for 7 d; 1st experimental diet for 28 d; control for 28 d (washout); 2nd experimental diet for 28 d.	*n* 10 (/10)<br>C: 221 ± 35 g (~39%)<br>P: 113 ± 19 g<br>F: 101 ± 18 g<br>E: 2261 ± 370 kcal	*n* 10 (/10)<br>C: 363 ± 76 g (~68%)<br>P: 106 ± 20 g<br>F: 24 ± 5 g<br>E: 2124 ± 418 kcal
Chantelau 1982 [21]	FLCD	Prior to intervention (i.e. baseline/comparator), patients had been educated on American Diabetes Association "conventional" diabetes diet, whereby 50% of total energy intake is from carbohydrate.	*n* 10<br>C: 156 ± 46 g (34 ± 5%)<br>P: 15 ± 2%<br>F: 51 ± 5%<br>E: 1300 – 3800 kcal	Baseline
Knight 2016 [23]	FLCD	DAFNE program at Queensland Diabetes Centre. Prior to intervention (i.e., baseline/comparator), "Participants often reported management that was based on a prescribed carbohydrate portion meal plan, and many were advised to consume carbohydrate at all meals and snacks."	*n* 46<br>C: 162 (143-204) g<br>(42 ± 7%)<br>P: 21 ± 4%<br>F: 34 ± 7%<br>E: 1592 (1378-1940) kcal	*n* 46<br>C: 198 (163-238) g<br>(42 ± 7%)<br>P: 21 ± 3%<br>F: 32 ± 7%<br>E: 1799 (1521-1931) kcal
Bernstein 1980[c] [26]	TLCD	Total elimination of simple sugars and fruits. Carbohydrate taken as vegetables, salad dressing or associated with high- protein packaged foods. Three main meals, without snacks.	*n* 1<br>C: 15%<br>P: ≥45%<br>F: ≤40%	Baseline
Ireland 1992 [22]	TLCD	Royal Melbourne Hospital outpatients. 'Present diet' formed the control diet whereby "…most had been attempting to increase their intake of complex carbohydrate and dietary fibre."	*n* 8<br>C: ~87 g (22 ± 2%)<br>P: 62 ± 1%<br>F: 16 ± 1%<br>E: 1617 ± 109 kcal	*n* 8<br>C: ~188 g (46 ± 1%)<br>P: 19 ± 1%<br>F: 33 ± 2%<br>E: 1627 ± 126 kcal
Krebs 2016 [10]	TLCD	All those referred to carbohydrate counting education to the Endocrine, Diabetes and Research Centre were approached to participate.	*n* 5 (/10)<br>C: 103 ± 22 g (~30%)<br>P: 76 ± 16 g<br>F: 69 ± 13 g<br>E: 1391 ± 159 kcal	*n* 5 (/10)<br>C: 203 ± 92 g (~44%)<br>P: 85 ± 42 g<br>F: 77 ± 15 g<br>E: 1854 ± 551 kcal

S5 Table: Reported Dietary Intake Data of Participants in Intervention and Comparator Groups of Included Studies

Nielsen 2012[c] [8]	TLCD	All individuals attending the education course from the start of 2004 to 2006 and adhering to it for at least the first 4 weeks are reported.	*n* 48<br>C: ≤ 75 g (15-20%)<br>P: 30%<br>F: 50-55%	Baseline
O'Neill 2003[c] [24]	VLCKD	Multi-component program that patients followed on their own. The charts selected for this study were based on assessment of the patients' ability to comply with the regimen.	*n* 10<br>C: 30 g	Baseline
Vernon 2003[c] [25]	VLCKD	Aim of initial diet was to produce detectable urinary ketones and achieve glycemic control. Maintenance diet individually determined by the highest level of carbohydrate where there were no urinary ketones.	*n* 1<br>C: 20-50 g	Baseline

Abbreviations: g (grams), d (day/s), *n* (sample size that completed intervention or control), C (total daily dietary carbohydrate), P (total daily dietary protein), F (total daily dietary fat), E (total daily energy intake), % (percent of total energy intake), DAFNE (Dose Adjustment for Normal Eating), baseline (dietary intake of participants before the intervention; data not reported in study).

a: Classification of intervention: False low-carbohydrate diet (FLCD) includes daily dietary carbohydrate above 130 g/d or 26% total energy intake; true low-carbohydrate diet (TLCD) includes daily dietary carbohydrate of 50-130 g/d; very low-carbohydrate ketogenic diet (VLCKD) includes daily dietary carbohydrate below 50 g/d.

b: Mean daily macronutrient and energy intakes for each group reported as $\bar{x} \pm$ SD or $\bar{x}$ (range) (to nearest whole unit). ~ indicates that value was calculated using Atwater factors and not taken from study (i.e., not reported).

c: Dietary intake of participants was not reported. Macronutrient and energy values are given as per the intervention prescription of the study.
