# Supplementary material for: Low-carbohydrate diets for type 1 diabetes mellitus: A systematic review
Source: PLoS One. 2018 Mar 29;13(3):e0194987. doi: 10.1371/journal.pone.0194987 (PMC5875783; doi:10.1371/journal.pone.0194987)
Supplement: S6 Table — (PDF) [file pone.0194987.s007.pdf]

S6 Table: Summary of risk of bias assessments for included randomised controlled trials using the Cochrane Collaboration's Risk of Bias for Randomised Controlled Trials assessment tool

Anderson 1991 [20]	Krebs 2016 [10]	
?	+	Random sequence generation (selection bias)
?	+	Allocation concealment (selection bias)
+	+	Blinding of participants (performance bias)
?	+	Blinding of personnel (performance bias)
+	+	Blinding of outcome assessment (detection bias)
+	+	Incomplete outcome data (attrition bias)
?	?	Selective outcome reporting (reporting bias)
+	+	Other Bias

Available options for judgement included 'low', 'high' or 'unclear' (risk of bias).

+ = Low risk of bias − = high risk of bias ? = unclear risk of bias
