# Supplementary material for: Low-carbohydrate diets for type 1 diabetes mellitus: A systematic review
Source: PLoS One. 2018 Mar 29;13(3):e0194987. doi: 10.1371/journal.pone.0194987 (PMC5875783; doi:10.1371/journal.pone.0194987)
Supplement: S7 Table — (PDF) [file pone.0194987.s008.pdf]

S7 Table: Risk of bias assessment for Anderson et al. (1991) [20]using the Cochrane Collaboration's Risk of Bias for Randomised Controlled Trials assessment tool

Domain	Judgment[a]	Support for judgment
**1. Random sequence generation** (selection bias)	Unclear	*Quote:* "they were then randomly assigned to either the LCLF or HCHF diet for 28 d" *Comment:* Randomised, yet no method reported so we cannot be sure if sequence generation is adequate.
**2. Allocation concealment** (selection bias)	Unclear	*Quote:* "random allocation, crossover design" *Comment:* Randomised, yet no method reported so we cannot be sure if allocation concealment is adequate.
**3. Blinding of participants** (performance bias)	Low	*Comment:* No blinding (nature of most diet studies), however the outcome is unlikely to be influenced due to its high objectivity (laboratory measurement). If the outcome was self-report HbA1c then knowledge of the allocated intervention may have had an influence on the outcome and bias for this domain would be 'high'.
**Blinding of personnel** (performance bias)	Unclear	*Comment:* Insufficient information.
**4. Blinding of outcome assessment** (detection bias)	Low	*Quote:* "Glycosylated haemoglobin was measured weekly by using the Bio-Rad Hemoglobin A1c Micro Column test method (Bio Rad, Hercules, CA)" *Comment:* No information on blinding, however the outcome (HbA1c) is unlikely to be affected due to the its objectivity as indicated by the transparency in the reporting of this outcome's measurement. The method of measurement appears highly objective. If the measurement was taken in the researchers' own laboratory and/or there was no clear information on the method of measurement, then a lack of blinding would raise bias for this domain to 'high'.
**5. Incomplete outcome data** (attrition bias)	Low	*Quote:* "Glycosylated haemoglobin values on subjects 1 and 4 were not included because of a change in method from an electrophoretic to a column technique. Values for these two subjects averaged 11.4% on the LCLF diet and 9.5% on the HCHF diet." *Comment:* There was incomplete outcome data for HbA1c (i.e., not all subjects results were included in the mean value), however a sufficient rationale was provided (along with the excluded results).
**6. Selective outcome reporting** (reporting bias)	Unclear	*Comment*: Insufficient information (no access to protocol).
**7. Other bias**	Low	*Comment:* The study appears to be free from other sources of bias.

Abbreviations: LCLF (low-carbohydrate, low fibre), HCHF (high-carbohydrate, high fibre), d (day/s).
a: Available options for judgement included 'low', 'high' or 'unclear' (risk of bias)
