# Supplementary material for: Low-carbohydrate diets for type 1 diabetes mellitus: A systematic review
Source: PLoS One. 2018 Mar 29;13(3):e0194987. doi: 10.1371/journal.pone.0194987 (PMC5875783; doi:10.1371/journal.pone.0194987)
Supplement: S8 Table — (PDF) [file pone.0194987.s009.pdf]

S8 Table: Risk of bias assessment for Krebs et al. (2016) [10] using the Cochrane Collaboration's Risk of Bias for Randomised Controlled Trials assessment tool

Domain	Judgement[a]	Support for judgment
1. **Random sequence generation** (selection bias)	Low	*Quote:* "Randomisation was achieved by computer generated random numbers put into sealed envelopes." *Comment:* Method of randomisation is reported and it is adequate.
2. **Allocation concealment** (selection bias)	Low	*Quote:* "Randomisation was achieved by computer generated random numbers put into sealed envelopes. Envelopes were then given out consecutively as participants enrolled." *Comment:* Method of allocation is reported and it is adequate.
3. **Blinding of participants (**performance bias)	Low	*Quote:* "Researchers collecting and analysing data were blinded to allocation, but it was not possible to blind participants." *Comment:* No blinding (nature of most diet studies), however the outcome is unlikely to be influenced due to its high objectivity (laboratory measurement). If the outcome was self-report HbA1c then knowledge of the allocated intervention may have had an influence on the outcome and bias for this domain would be 'high'.
**Blinding of personnel** (performance bias)	Low	*Quote:* "Researchers collecting and analysing data were blinded to allocation"
4. **Blinding of outcome assessment*** (detection bias)	Low	*Quote:* "A venous blood sample was taken to analyse HbA1c". "All blood samples were analysed on completion of the study using standard commercial assays (Roche Diagnostics New Zealand) by an accredited laboratory (Diabetes and Lipid Laboratory, University of Otago, Dunedin, New Zealand)." *Comment:* Outcome measurement taken by third party laboratory, so a lack of blinding assessment is unlikely to influence result.
5. **Incomplete outcome data** (attrition bias)	Low	*Comment:* No missing outcome data. *Quote:* "Ten participants … entered and completed the study."
6. **Selective outcome reporting** (reporting bias)	Unclear	*Comment:* No information (no access to protocol).
7. **Other bias**	Low	*Comment:* Study appears to be free from other sources of bias.

a: Available options for judgement included 'low', 'high' or 'unclear' (risk of bias).
