# Supplementary material for: Low-carbohydrate diets for type 1 diabetes mellitus: A systematic review
Source: PLoS One. 2018 Mar 29;13(3):e0194987. doi: 10.1371/journal.pone.0194987 (PMC5875783; doi:10.1371/journal.pone.0194987)
Supplement: S9 Table — (PDF) [file pone.0194987.s010.pdf]

S9 Table: Summary of Quality Assessments Using the National Institute of Health’s Quality Assessment Tool for Pre-Post Intervention Studies with No Control Group

Chantelau 1982 [21]	Ireland 1992 [22]	Knight 2016 [23]	Nielsen 2012 [8]	
+	+	+	+	Study question / objective
−	+	+	+	Eligibility criteria and study population
+	+	+	+	Study participants representative of clinical populations of interest
?	−	?	?	All eligible participants enrolled
+	−	+	+	Sample size
+	+	+	+	Intervention clearly described
+	+	?	+	Outcome measures clearly described, valid, and reliable
?	?	?	−	Blinding of outcome assessors
+	+	−	+	Follow-up rate
+	+	+	+	Statistical analysis
−	−	−	+	Multiple outcome measures
?	?	?	+	Group-level interventions and individual-level outcome efforts

Available judgements for supporting criteria (items 1-12) include ‘yes’, ‘no’ and ‘other’.

+ = Yes (Good Quality) − = No (Poor Quality)

? = Unclear (cannot determine, not applicable, not reported)
