# Supplementary material for: Low-carbohydrate diets for type 1 diabetes mellitus: A systematic review
Source: PLoS One. 2018 Mar 29;13(3):e0194987. doi: 10.1371/journal.pone.0194987 (PMC5875783; doi:10.1371/journal.pone.0194987)
Supplement: S10 Table — (PDF) [file pone.0194987.s011.pdf]

S10 Table: Quality assessment for Chantelau et al. (1982) [21] using The National Institute of Health's Quality Assessment Tool for Pre-Post Intervention Studies with No Control Group

Criteria	Judgement[a]	Support
1. Was the study question or objective clearly stated?	Yes	*Quote:* "This study was performed to evaluate the effects of a limited liberalization of diabetes diet on blood glucose control and serum lipid levels in type 1 diabetic patients under conditions of CSII"
2. Were eligibility/selection criteria for the study population pre-specified and clearly described?	No	*Quote:* "Ten patients with type I diabetes mellitus were studies after obtaining their informed consent."<br>*Comment:* Study lists the clinical data of included patients in a table but does not state a who, when or where for this study population.
3. Were the participants in the study representative of those who would be eligible for the intervention in the general or clinical population of interest?	Yes	*Comment:* All participants have type 1 diabetes and were working or studying adults (18-34 years) with a mean diabetes duration of 14 years. This group represented a "highly motivated group".
4. Were all eligible participants that met the pre-specified entry criteria enrolled?	Other (CD)	*Comment:* There is no clear criteria for inclusion (item 2) so this item cannot be determined.
5. Was the sample size sufficiently large to provide confidence in the findings?	Yes	*Comment:* The authors did not provide reasons for recruiting the number of individuals analysed. However, a statistically significant effect (in HbA1c) was detected with this sample size (indicated by $P < 0.0025$) so it appears adequate for this outcome.
6. Was the intervention clearly described and delivered consistently across the study population?	Yes	*Quote:* "All patients participating in the study were allowed to choose between the conventional diabetes diet and the 'less restricted diabetes diet'".<br>*Comment:* The intervention was more self-directed then 'delivered' (at least for the phase we are interested in). The given description of "increased dietary freedom" was consistently delivered across the population, even though this particular intervention would be carried out highly individually.
7. Were the outcome measures pre-specified, clearly defined, valid, reliable, and assessed consistently across all study participants?	Yes	*Quote:* "At onsent and at the end of the study period, HbA1c … were determined using methods described elsewhere"<br>*Comment:* The method reference provided a clear definition of the method, which appeared valid and reliable (Fluckiger, R., and Winterhalter, K. H.: In vitro synthesis of hemoglobin A,,. FEBS Lett. 71: 356-63, 1976.). All participants were assessed consistently.
8. Were the people assessing the outcomes blinded to the participants' interventions?	Other (NR)	*Comment:* No information on blinding specifically or on who was responsible for assessing the outcome (HbA1c), only the method is described.
9. Was the loss to follow-up after baseline 20% or less? Were those lost to follow-up accounted for in the analysis?	Yes	*Comment:* Follow-up was 100%. There was no missing outcome data.

S10 Table: Quality assessment for Chantelau et al. (1982) [21] using The National Institute of Health's Quality Assessment Tool for Pre-Post Intervention Studies with No Control Group

10. Did the statistical methods examine changes in outcome measures from before to after the intervention? Were statistical tests done that provided p values for the pre-to-post changes?	Yes	*Comment:* The study reports the statistical significance of the outcome (HbA1c) from before to after the intervention ($P < 0.0025$).
11. Were outcome measures of interest taken multiple times before the intervention and multiple times after the intervention?	No	*Quote:* "At onsent and at the end of the study period, HbA1c … were determined"<br>*Comment:* It appears that outcome measurements were only taken at one time-point before and one time point after the intervention.
12. If the intervention was conducted at a group level did the statistical analysis take into account the use of individual-level data to determine effects at the group level?	Other (NA)	*Comment:* Intervention was not conducted at a group level.
Overall Rating	**Fair**	*Additional support (comment):* This study performed well in 7 items of this assessment tool and there was an attempt to control for the major confounder, insulin. All participants were set up on CSII before the study started. However, the lack of a clear inclusion/exclusion criteria for participants chosen for the study introduces potential for bias.

Abbreviations: CSII (continuous subcutaneous insulin infusion).

a: Available judgements for supporting criteria (items 1-12) include 'yes', 'no' and 'other'. 'Other' should be specified as CD (cannot determine), NA (not applicable) or NR (not reported). Available judgements for overall rating include 'good', 'fair' or 'poor'.
