# Supplementary material for: Low-carbohydrate diets for type 1 diabetes mellitus: A systematic review
Source: PLoS One. 2018 Mar 29;13(3):e0194987. doi: 10.1371/journal.pone.0194987 (PMC5875783; doi:10.1371/journal.pone.0194987)
Supplement: S11 Table — (PDF) [file pone.0194987.s012.pdf]

S11 Table: Quality assessment for Ireland et al. (1992) [22] using The National Institute of Health's Quality Assessment Tool for Pre-Post Intervention Studies with No Control Group

| Criteria | Judgement[a] | Support |
|---|---|---|
| 1. Was the study question or objective clearly stated? | Yes | *Quote:* "The aim of this study was to determine whether IDDM has a similar metabolic response to NIDDM when following two low-carbohydrate diets that differ in fat content." |
| 2. Were eligibility/selection criteria for the study population pre-specified and clearly described? | Yes | *Quote:* "Thirteen patients were recruited from the outpatient clinic of the Royal Melbourne Hospital." "All subjects met the composite clinical criteria of IDDM, namely, being <40 yr of age and being <120% of desirable body weight at the time of diagnosis, and having been placed on insulin therapy within 2 yr of diagnosis." |
| 3. Were the participants in the study representative of those who would be eligible for the intervention in the general or clinical population of interest? | Yes | Comment: All participants have type 1 diabetes (8 women, 5 men, age 30.3 ± 1.6 yr, BMI 23.1 ± 0.6) (mean ± SE). Diabetes duration ranged from 2 to 35 years. |
| 4. Were all eligible participants that met the pre-specified entry criteria enrolled? | No | *Quote:* "…only those subjects with the lowest energy requirements were invited to take part in this phase (low-fat, low-carbohydrate diet) of the experiment" *Comment:* Only 5 subjects took part in the entire study (two experimental diets), another 5 followed one experimental diet only, and the remaining 3 followed the other experimental diet only. It seems that additional criteria were applied after participants were included in the study which prevented all participants from completing the entire study. |
| 5. Was the sample size sufficiently large to provide confidence in the findings? | No | *Comment:* Sample size ($n$ = 8) was not sufficient to detect an effect in the primary outcome (HbA1c). The follow-up time (2 weeks) was also insufficient as HbA1c is most effective at detecting changes in blood sugar over 3-months. However, a significant effect was detected in a secondary outcome (insulin). |
| 6. Was the intervention clearly described and delivered consistently across the study population? | Yes | *Comment:* The intervention we are interested in for this review (low-fat, low-carbohydrate diet) was delivered consistently across the population, with all major meals provided to participants. |
| 7. Were the outcome measures pre-specified, clearly defined, valid, reliable, and assessed consistently across all study participants? | Yes | *Quote*: "The Coming Glytrac kit (Palo Alto, CA) was used to measure total GHb". |
| 8. Were the people assessing the outcomes blinded to the participants' interventions? | Other (NR) | *Comment:* No information on blinding specifically or on who was responsible for assessing the outcome (HbA1c), only the method is described. |
| 9. Was the loss to follow-up after baseline 20% or less? Were those lost to follow-up accounted for in the analysis? | Yes | *Comment:* No missing outcome data. Eight participants completed intervention and outcome data for 8 participants included in results. |

S11 Table: Quality assessment for Ireland et al. (1992) [22] using The National Institute of Health's Quality Assessment Tool for Pre-Post Intervention Studies with No Control Group

| | | |
|---|---|---|
| 10. Did the statistical methods examine changes in outcome measures from before to after the intervention? Were statistical tests done that provided p values for the pre-to-post changes? | Yes | *Comment*: The study reported the statistical significance of the outcomes from before to after the intervention if they were significant ($P < 0.05$). A P-value was not reported for change in HbA1c, however the change was indicated as non-significant. |
| 11. Were outcome measures of interest taken multiple times before the intervention and multiple times after the intervention? | No | *Comment:* It appears that outcome measurements were only taken at one time point before and one time point after the intervention. |
| 12. If the intervention was conducted at a group level did the statistical analysis take into account the use of individual-level data to determine effects at the group level? | Other (NA) | *Comment:* Intervention was not conducted at a group level. |
| ***Overall Rating*** | **Poor** | *Additional support (comment):* There was no attempt to control for a major confounder, insulin. |

Abbreviations: IDDM (insulin dependent diabetes mellitus), NIDDM (non-insulin dependent diabetes mellitus), SE (standard error), GHb (glycosylated haemoglobin).

a: Available judgements for supporting criteria (items 1-12) include 'yes', 'no' and 'other'. 'Other' should be specified as CD (cannot determine), NA (not applicable) or NR (not reported). Available judgements for overall rating include 'good', 'fair' or 'poor'.
