# Supplementary material for: Low-carbohydrate diets for type 1 diabetes mellitus: A systematic review
Source: PLoS One. 2018 Mar 29;13(3):e0194987. doi: 10.1371/journal.pone.0194987 (PMC5875783; doi:10.1371/journal.pone.0194987)
Supplement: S12 Table — (PDF) [file pone.0194987.s013.pdf]

S12 Table: Quality assessment for Knight et al. (2016) [23] using The National Institute of Health's Quality Assessment Tool for Pre-Post Intervention Studies with No Control Group

Criteria	Judgement[a]	Support
1. Was the study question or objective clearly stated?	Yes	*Quote:* "The aim of this study is to observe and quantify the impact of flexible insulin therapy education with an overt promotion of increased dietary freedom, on energy, macronutrient and fat intake in adults with T1DM and achievement of EASD nutrient recommendations."
2. Were eligibility/selection criteria for the study population pre-specified and clearly described?	Yes	*Quote:* "Dietary assessment was performed alongside standard data collection procedures in all participants attending the DAFNE programme at the Queensland Diabetes Centre, Mater Health Services, Brisbane, Australia for the first two years of course delivery. Eligibility criteria for DAFNE participation has previously been described (reference provided). The following exclusion criteria were applied to the dietary data: pregnancy or clinically diagnosed eating disorder, either pre-existing or occurring within 12 months of course participation."
3. Were the participants in the study representative of those who would be eligible for the intervention in the general or clinical population of interest?	Yes	*Comment:* All participants have type 1 diabetes (29-51 years of age, BMI of 23-28 and baseline HbA1c of 7.1-8.5%). Diabetes duration was 11-26 years.
4. Were all eligible participants that met the pre-specified entry criteria enrolled?	Other (CD)	*Comment:* There is insufficient information to determine whether all participants who met the eligibility criteria were enrolled in the study.
5. Was the sample size sufficiently large to provide confidence in the findings?	Yes	*Comment:* Sample size is a potential weakness of the audit, with 46 participants completing the study and no significant change in mean HbA1c detected. However, sufficient follow-up time was allowed (12 months) and it is likely that the intervention may not have been effective in reducing HbA1c to levels beyond what the participants had achieved at baseline.
6. Was the intervention clearly described and delivered consistently across the study population?	Yes	*Quote:* "The Dose Adjustment for Normal Eating (DAFNE) programme is a flexible insulin therapy programme, which actively promoted a 'freedom to eat' concept, with the motto, 'Eat what you like, like what you eat' when initially introduced to the United Kingdom and Australia. Education relating to healthy eating was included in the OzDAFNE structured curriculum, however the focus of the programme was the acquisition of patient-based skills in insulin adjustment. Healthy eating was addressed only briefly and dietary prescription of macronutrient intake was not addressed."
7. Were the outcome measures pre-specified, clearly defined, valid, reliable, and assessed consistently across all study participants?	Other (NR)	*Comment:* Outcomes of the study were dietary data, weight loss (in the 44 participants who intended to lose weight), and frequency of severe hypoglycaemia. HbA1c was able to be obtained from the author, yet was

S12 Table: Quality assessment for Knight et al. (2016) [23] using The National Institute of Health's Quality Assessment Tool for Pre-Post Intervention Studies with No Control Group

		not reported in the study as an outcome. HbA1c was assessed according to standard DAFNE procedures which we assume to be valid. It is not clear how other outcomes were measured and whether they were self-report, however, this item is judged according to HbA1c.
8. Were the people assessing the outcomes blinded to the participants' interventions?	Other (NR)	*Comment:* Unlikely. There is no information on blinding, however all participants received the same intervention.
9. Was the loss to follow-up after baseline 20% or less? Were those lost to follow-up accounted for in the analysis?	No	*Comment:* 29/75 (39%) participants were lost to follow-up. The baseline characteristics of those who attended at 12 months post course compared to those who were lost to follow-up is reported in the study. Those lost to follow-up were not included in the results for HbA1c.
10. Did the statistical methods examine changes in outcome measures from before to after the intervention? Were statistical tests done that provided p values for the pre-to-post changes?	Yes	*Comment:* P-values were calculated and provided for reported outcomes.
11. Were outcome measures of interest taken multiple times before the intervention and multiple times after the intervention?	No	*Comment:* It appears that outcome measurements were only taken at one time point before and one time point after the intervention.
12. If the intervention was conducted at a group level did the statistical analysis take into account the use of individual-level data to determine effects at the group level?	Other (CD)	*Comment:* It is not clear whether patients attended the DAFNE program as a group or individually.
Overall Rating	**Poor**	*Additional support (comment):* This study did not attempt to control for a major confounder, insulin. Insulin flexibility was encouraged by design (a component of the DAFNE program).

Abbreviations: T1DM (type 1 diabetes mellitus), EASD (European Association for the Study of Diabetes), DAFNE (Dose Adjustment for Normal Eating).

a: Available judgements for supporting criteria (items 1-12) include 'yes', 'no' and 'other'. 'Other' should be specified as CD (cannot determine), NA (not applicable) or NR (not reported). Available judgements for overall rating include 'good', 'fair' or 'poor'.
