# Supplementary material for: Low-carbohydrate diets for type 1 diabetes mellitus: A systematic review
Source: PLoS One. 2018 Mar 29;13(3):e0194987. doi: 10.1371/journal.pone.0194987 (PMC5875783; doi:10.1371/journal.pone.0194987)
Supplement: S13 Table — (PDF) [file pone.0194987.s014.pdf]

S13 Table: Quality assessment for Nielsen et al. (2012) [8] using The National Institute of Health's Quality Assessment Tool for Pre-Post Intervention Studies with No Control Group

Criteria	Judgment[a]	Support
1. Was the study question or objective clearly stated?	Yes	*Quote:* "The purpose is to map adherence among the individuals with type 1 diabetes, who attended the educational course at least 4 years earlier."
2. Were eligibility/selection criteria for the study population pre-specified and clearly described?	Yes	*Quote:* "All individuals with HbA1c ≥ 6.1% (DCCT: 7.1%) attending the educational course from the start of 2004 to 2006 and adhering to it for at least the first 4 weeks are reported here." For inclusion to the course itself, "All individuals attending the educational course here have sought it themselves after having had written information of the regimen."<br>*Comment:* Inclusion criteria for this study includes information on who, when and where of the study population.
3. Were the participants in the study representative of those who would be eligible for the intervention in the general or clinical population of interest?	Yes	*Quote:* "Altogether 48 persons with HbA1c ≥ 6.1% (DCCT: 7.1%) had attended the course, 31 women and 17 men. Most had attended diabetes education before in the form of diabetes schools, diabetes camps etc. Fourteen used insulin pumps. There was no relation between the use of the pumps and HbA1c. Mean age and diabetes duration (± SD) was 52 ± 11.5 years and 24 ± 12 years respectively."
4. Were all eligible participants that met the pre-specified entry criteria enrolled?	Other (CD)	*Comment:* There is insufficient information to determine whether all participants who met the eligibility criteria were enrolled in the study.
5. Was the sample size sufficiently large to provide confidence in the findings?	Yes	*Comment:* A statistically significant effect (in HbA1c) was detected with this sample size (n = 45) indicated by $P < 0.001$) so it appears adequate for this outcome.
6. Was the intervention clearly described and delivered consistently across the study population?	Yes	*Comment:* A supplementary file that describes the intervention in sufficient detail is provided online. All participants received the same information, however it was up to them individually to make the necessary changes.
7. Were the outcome measures pre-specified, clearly defined, valid, reliable, and assessed consistently across all study participants?	Yes	*Quote:* "HbA1c measurements were done in the same laboratory using the Mono-S method used in Sweden."
8. Were the people assessing the outcomes blinded to the participants' interventions?	Other (NR)	*Comment:* Unlikely. There is no information on blinding, however all participants received the same intervention.
9. Was the loss to follow-up after baseline 20% or less? Were those lost to follow-up accounted for in the analysis?	Yes	*Comment:* All patients who attended the course (n = 48) were followed-up at 4 years.
10. Did the statistical methods examine changes in outcome measures from before to after the intervention? Were statistical tests done that provided p values for the pre-to-post changes?	Yes	*Comment:* P-values were calculated and provided for reported outcomes.

S13 Table: Quality assessment for Nielsen et al. (2012) [8] using The National Institute of Health's Quality Assessment Tool for Pre-Post Intervention Studies with No Control Group

11. Were outcome measures of interest taken multiple times before the intervention and multiple times after the intervention?	Yes	*Quote:* "HbA1c after 3 months shows the effect of short-time adherence. From then on we have assessed adherence by comparing individual HbA1c values with the start and 3-months values."<br>*Comment:* HbA1c was taken every 3 months for 4 years after the intervention.
12. If the intervention was conducted at a group level did the statistical analysis take into account the use of individual-level data to determine effects at the group level?	Yes	*Comment:* The intervention was delivered in small groups, however individual-level data was used to determine effects.
Overall Rating	**Fair**	*Additional support (comment):* There was a very good attempt to control for insulin in this study by devising a specific insulin protocol for all participants to follow (details provided in supplementary material of report). Despite this report being a retrospective case-series, all participants exposed to the intervention were included in the results (including non-adherers).

Abbreviations: DCCT (the Diabetes Control and Complications Trial), SD (standard deviation).

a: Available judgements for supporting criteria (items 1-12) include 'yes', 'no' and 'other'. 'Other' should be specified as CD (cannot determine), NA (not applicable) or NR (not reported). Available judgements for overall rating include 'good', 'fair' or 'poor'.
