# Supplementary material for: Low-carbohydrate diets for type 1 diabetes mellitus: A systematic review
Source: PLoS One. 2018 Mar 29;13(3):e0194987. doi: 10.1371/journal.pone.0194987 (PMC5875783; doi:10.1371/journal.pone.0194987)
Supplement: S14 Table — (PDF) [file pone.0194987.s015.pdf]

S14 Table: Risk of Bias Assessment for Case-Series using Joanna Brigg's Critical Appraisal Tool for Case-Series

Item	Vernon 2003 [25]	O'neill 2003 [24]
	Judgement[a]	
1. Were there clear criteria for inclusion in the case series?	Yes	Yes
2. Was the condition measured in a standard, reliable way for all participants included in the case series?	Unclear	Unclear
3. Were valid methods used for identification of the condition for all participants included in the case series?	Yes	Yes
4. Did the case series have consecutive inclusion of participants?	No	No
5. Did the case series have complete inclusion of participants?	No	No
6. Was there clear reporting of the demographics of the participants in the study?	Yes	No
7. Was there clear reporting of clinical information of the participants?	No	No
8. Were the outcomes or follow up results of cases clearly reported?	Yes	Yes
9. Was there clear reporting of the presenting site(s)/clinic(s) demographic information?	No	No
10. Was statistical analysis appropriate?	Yes	Yes
Overall Appraisal	**Low risk**	**High risk**

a: Available judgments for each supporting item were 'yes', 'no', 'unclear' and 'not applicable'.
