# Supplementary material for: Low-carbohydrate diets for type 1 diabetes mellitus: A systematic review
Source: PLoS One. 2018 Mar 29;13(3):e0194987. doi: 10.1371/journal.pone.0194987 (PMC5875783; doi:10.1371/journal.pone.0194987)
Supplement: S15 Table — (PDF) [file pone.0194987.s016.pdf]

S15 Table. Risk of Bias Assessment for Bernstein (1980) [26] using Joanna Brigg's Critical Appraisal Tool for Case-Reports

Item	Judgement[a]
1. Were patient's demographic characteristics clearly described?	Yes
2. Was the patient's history clearly described and presented as a timeline?	No
3. Was the current clinical condition of the patient on presentation clearly described?	Yes
4. Were diagnostic tests or assessment methods and the results clearly described?	Yes
5. Was the intervention(s) or treatment procedure(s) clearly described?	Yes
6. Was the post-intervention clinical condition clearly described?	Yes
7. Were adverse events (harms) or unanticipated events identified and described?	Yes
8. Does the case report provide takeaway lessons?	Yes
Overall Appraisal	**Low risk**

a: Available judgments for each supporting item were 'yes', 'no', 'unclear' and 'not applicable'.
