# Supplementary material for: Low-carbohydrate diets for type 1 diabetes mellitus: A systematic review
Source: PLoS One. 2018 Mar 29;13(3):e0194987. doi: 10.1371/journal.pone.0194987 (PMC5875783; doi:10.1371/journal.pone.0194987)
Supplement: S16 Table — (PDF) [file pone.0194987.s017.pdf]

S16 Table. Risk of Bias Assessment for Chantelau et al. (1982) [21] using ROBINS-E[a]

Domain / Support	Judgement[b]	Additional Notes
Domain 1. Bias due to confounding	**Moderate**	
• Is there potential for confounding of the effect of exposure in this study?	Yes	Insulin treatment is a major confounder.
• Was the analysis based on splitting follow up time according to exposure received?	No	-
• Did the authors use an appropriate analysis method that adjusted for all the critically important confounding areas (at baseline)?	Yes	All participants placed on CSII 4 to 5 weeks before the study started.
• Were confounding areas that were adjusted for measured validly and reliably by the variables available in this study?	No information	It is assumed that CSII therapy effectively measured insulin. However, doses were not reported.
• Did the authors avoid adjusting for post-exposure variables?	Yes	There was no specific adjustment to insulin protocol post-exposure.
Domain 2. Bias in the selection of participants into the study	**Low**	
• Was selection of participants into the study (or into the analysis) based on variables related to either the exposure or the outcome? (measured after the start of the exposure)	No	I.e., variables related to carbohydrate intake or HbA1c.
• Do start of follow-up and start of exposure coincide for most participants?	Yes	The low-carbohydrate intake was an observed exposure coinciding with the start of the educational intervention.
Domain 3. Bias in the classification of exposures	**Low**	
• Is exposure status well defined?	Yes	Mean carbohydrate intake of participants in grams per day with standard deviations was reported.
• Did entry into the cohort begin with start of the exposure?	Yes	Likely. Prior to study, participants were following ADA's diabetes diet (50% energy as carbohydrate).
• Could classification of exposure status have been affected by knowledge of the outcome or risk of the outcome?	No	Unlikely.
• Are the levels, duration, or range of exposure of the population at risk sufficient or adequate to detect an effect of exposure?	Yes	Duration: 4-6 months. Level of exposure (mean ± SD): 156 ± 46 g (34 ± 5%). Mean level is adequate however, participants at higher end of carbohydrate intake range may not have had adequate levels of exposure to detect an effect.
• Is the follow-up period adequate to allow for the development of the outcome of interest?	Yes	4-6 months is sufficient for HbA1c.

S16 Table. Risk of Bias Assessment for Chantelau et al. (1982) [21] using ROBINS-E[a]

• Were exposure methods robust (including methods used to input data)?	Yes	Seven patients were visited at home or work over 3 days. Food intake (including quantities) was recorded. However, 3 participants did not have their food intake measured.
Domain 4. Bias due to departures from intended exposures	**Moderate**	
• Is there concern that changes in exposure status occurred among participants?	Yes	Intervention was "less restricted diabetes diet" so it is unlikely that exposure status was stable.
• Were adjustment techniques used that are likely to correct for these issues?	No	Food intake only recorded over 3 consecutive days. It may would been more appropriate to measure exposure over 3 non-consecutive days spread over the 4-5 months.
Domain 5. Bias due to missing data	**Low**	
• Was there missing outcome data?	No	HbA1c data available for n = 10.
• Are the proportion of participants and reasons for missing data similar across exposures?	-	-
• Were appropriate statistical methods used to account for missing data?	-	-
Domain 6. Bias in measurement of outcomes	**Moderate**	
• Could the outcome measure have been influenced by knowledge of the exposure received?	No	HbA1c is an objective outcome.
• Was the outcome measure sensitive?	Yes	A reference was provided for details on the method of outcome measurement. The method was clearly defined and appeared valid and reliable.
• Were outcome assessors unaware of the exposure received by study participants?	No information	-
• Were the methods of outcome assessment comparable across exposure groups?	-	Not appropriate (single group).
Domain 7. Bias in selection of the reported result	**No Information**	
• Are reported effect estimate(s) likely to be selected on the basis of results from (i) multiple outcome measurements within the outcome domain, (ii) multiple analyses of the exposure-outcome relationship, or (iii) different subgroups	NI	Due to nature of non-RCT nutrition studies not publishing their protocols, this domain must be judged as "no information".
Overall Risk of Bias	**Moderate**	The highest judgement in any domain.

Abbreviations / symbols: - (item left blank if not appropriate and/or supporting text not required), CSII (continuous subcutaneous insulin infusion), ADA (American Diabetes Association), RCT (randomised controlled trial).

a: ROBINS-E (risk of bias in non-randomised studies of exposures) is a critical appraisal tool currently under development by the Cochrane Collaboration. For this study, the exposure was a low-carbohydrate diet (<45% total energy intake from dietary carbohydrate).

b: Available judgements for signalling questions include ‘yes’, ‘no’ and ‘no information’. Available judgements for risk of bias of individual domains and for overall risk of bias include ‘low’, ‘moderate’, ‘serious’, ‘critical’, ‘no information’.
