# Supplementary material for: Low-carbohydrate diets for type 1 diabetes mellitus: A systematic review
Source: PLoS One. 2018 Mar 29;13(3):e0194987. doi: 10.1371/journal.pone.0194987 (PMC5875783; doi:10.1371/journal.pone.0194987)
Supplement: S17 Table — (PDF) [file pone.0194987.s018.pdf]

S17 Table. Risk of Bias Assessment for Knight et al. (2016) [23] using ROBINS-E[a]

Domain / Support	Judgement[b]	Additional Notes
Domain 1. Bias due to confounding	**Critical**	
• Is there potential for confounding of the effect of exposure in this study?	Yes	Insulin treatment is a major confounder.
• Was the analysis based on splitting follow up time according to exposure received?	No	-
• Did the authors use an appropriate analysis method that adjusted for all the critically important confounding areas (at baseline)?	No	There was no attempt to control for insulin. Insulin flexibility encouraged.
• Were confounding areas that were adjusted for measured validly and reliably by the variables available in this study?	-	-
• Did the authors avoid adjusting for post-exposure variables?	Yes	-
Domain 2. Bias in the selection of participants into the study	**Low**	
• Was selection of participants into the study (or into the analysis) based on variables related to either the exposure or the outcome? (measured after the start of the exposure)	No	I.e., variables related to carbohydrate intake or HbA1c
• Do start of follow-up and start of exposure coincide for most participants?	Yes	The low-carbohydrate intake was an observed exposure coinciding with the start of the educational intervention.
Domain 3. Bias in the classification of exposures	**Moderate**	
• Is exposure status well defined?	Yes	Mean carbohydrate intake of participants in grams per day with standard deviations was reported.
• Did entry into the cohort begin with start of the exposure?	Yes	Pre and post course dietary data from diet histories, including carbohydrate intake, is reported.
• Could classification of exposure status have been affected by knowledge of the outcome or risk of the outcome?	No	Unlikely.
• Are the levels, duration, or range of exposure of the population at risk sufficient or adequate to detect an effect of exposure?	No	Unlikely. Carbohydrate was reduced by ~30 grams per day which was statistically significant. However, there was no change as %TEI.
• Is the follow-up period adequate to allow for the development of the outcome of interest?	Yes	12 months for HbA1c is sufficient.
• Were exposure methods robust (including methods used to input data)?	Yes	Specialist diabetes dietitian collected dietary data using an open-ended, interview administered diet-history.

S17 Table. Risk of Bias Assessment for Knight et al. (2016) [23] using ROBINS-E[a]

Domain 4. Bias due to departures from intended exposures	**Low**	
• Is there concern that changes in exposure status occurred among participants?	Yes	Intervention was "increased dietary flexibility" so it is unlikely that exposure status was stable.
• Were adjustment techniques used that are likely to correct for these issues?	Yes	A diet history captures overall intake over the follow-up period so it is likely that the dietitians captured potential exposure changes.
Domain 5. Bias due to missing data	**Moderate**	
• Was there missing outcome data?	No	Out of participants in exposure group of interest to our review (*n* = 46), outcome data (provided via email by B. Knight) was complete.
• Are the proportion of participants and reasons for missing data similar across exposures?	-	-
• Were appropriate statistical methods used to account for missing data?	-	-
Domain 6. Bias in measurement of outcomes	**Moderate**	
• Could the outcome measure have been influenced by knowledge of the exposure received?	No	HbA1c is an objective outcome.
• Was the outcome measure sensitive?	Yes	HbA1c was assessed according to standard DAFNE procedures which is assumed to be valid.
• Were outcome assessors unaware of the exposure received by study participants?	No information	Unlikely.
• Were the methods of outcome assessment comparable across exposure groups?	-	Not appropriate (single group).
Domain 7. Bias in selection of the reported result	**No Information**	
• Are reported effect estimate(s) likely to be selected on the basis of results from (i) multiple outcome measurements within the outcome domain, (ii) multiple analyses of the exposure-outcome relationship, or (iii) different subgroups	NI	Due to nature of non-RCT nutrition studies not publishing their protocols, this domain must be judged as "no information".
Overall Risk of Bias	**Critical**	The highest judgement in any domain.

Abbreviations / symbols: - (item left blank if not appropriate and/or supporting text not required), %TEI (percent total energy intake), RCT (randomised controlled trial).
a: ROBINS-E (risk of bias in non-randomised studies of exposures) is a critical appraisal tool currently under development by the Cochrane Collaboration. For this study, the exposure was a low-carbohydrate diet (<45% total energy intake from dietary carbohydrate).

b: Available judgements for signalling questions include ‘yes’, ‘no’ and ‘no information’. Available judgements for risk of bias of individual domains and for overall risk of bias include ‘low’, ‘moderate’, ‘serious’, ‘critical’, ‘no information’.
